# Machine learning-based prediction model for responses of bDMARDs in patients with rheumatoid arthritis and ankylosing spondylitis

**DOI:** 10.1186/s13075-021-02635-3

**Published:** 2021-10-09

**Authors:** Seulkee Lee, Seonyoung Kang, Yeonghee Eun, Hong-Hee Won, Hyungjin Kim, Jaejoon Lee, Eun-Mi Koh, Hoon-Suk Cha

**Affiliations:** 1 Department of Medicine, Samsung Medical Center, Sungkyunkwan University School of Medicine, 81 Irwon-ro, Gangnam-gu, Seoul, 06351 Republic of Korea; 2 Samsung Advanced Institute for Health Sciences & Technology (SAIHST), Sungkyunkwan University, Samsung Medical Center, Seoul, Republic of Korea

**Keywords:** Rheumatoid arthritis, Ankylosing spondylitis, Machine learning, TNFi

## Abstract

**Background:**

Few studies on rheumatoid arthritis (RA) have generated machine learning models to predict biologic disease-modifying antirheumatic drugs (bDMARDs) responses; however, these studies included insufficient analysis on important features. Moreover, machine learning is yet to be used to predict bDMARD responses in ankylosing spondylitis (AS). Thus, in this study, machine learning was used to predict such responses in RA and AS patients.

**Methods:**

Data were retrieved from the Korean College of Rheumatology Biologics therapy (KOBIO) registry. The number of RA and AS patients in the training dataset were 625 and 611, respectively. We prepared independent test datasets that did not participate in any process of generating machine learning models. Baseline clinical characteristics were used as input features. Responders were defined as those who met the ACR 20% improvement response criteria (ACR20) and ASAS 20% improvement response criteria (ASAS20) in RA and AS, respectively, at the first follow-up. Multiple machine learning methods, including random forest (RF-method), were used to generate models to predict bDMARD responses, and we compared them with the logistic regression model.

**Results:**

The RF-method model had superior prediction performance to logistic regression model (accuracy: 0.726 [95% confidence interval (CI): 0.725–0.730] vs. 0.689 [0.606–0.717], area under curve (AUC) of the receiver operating characteristic curve (ROC) 0.638 [0.576–0.658] vs. 0.565 [0.493–0.605], F1 score 0.841 [0.837–0.843] vs. 0.803 [0.732–0.828], AUC of the precision-recall curve 0.808 [0.763–0.829] vs. 0.754 [0.714–0.789]) with independent test datasets in patients with RA. However, machine learning and logistic regression exhibited similar prediction performance in AS patients. Furthermore, the patient self-reporting scales, which are patient global assessment of disease activity (PtGA) in RA and Bath Ankylosing Spondylitis Functional Index (BASFI) in AS, were revealed as the most important features in both diseases.

**Conclusions:**

RF-method exhibited superior prediction performance for responses of bDMARDs to a conventional statistical method, i.e., logistic regression, in RA patients. In contrast, despite the comparable size of the dataset, machine learning did not outperform in AS patients. The most important features of both diseases, according to feature importance analysis were patient self-reporting scales.

**Supplementary Information:**

The online version contains supplementary material available at 10.1186/s13075-021-02635-3.

## Background

Biologic disease-modifying antirheumatic drugs (bDMARDs) play a pivotal role in the treatment of various rheumatologic diseases, such as rheumatoid arthritis (RA) and ankylosing spondylitis (AS), particularly those resistant to conventional synthetic disease-modifying rheumatic drugs (csDMARDs). However, approximately 30% and 20% of RA [1, 2] and AS [3–5] patients, respectively, do not respond well to their initial bDMARD therapy. A few months are required to determine the efficacy of the medications. Non-responders could experience high drug costs, unimproved disease conditions, and side effects during this period [6–9]. Therefore, methods to predict the responses prior to the start of bDMARDs is garnering substantial interest.

Several studies simply identified and compared clinical factors, such as sex, age, disease duration, and disease activity, in both diseases to influence the treatment responses of bDMARDs [10, 11], rather than making a predictive model. Because the relationship between clinical variables and phenotypes is complex, machine learning methods outperform conventional statistical models in predicting clinical outcomes in various circumstances [12–15]. Recently, the use of machine learning to predict anti-tumor necrosis factor (TNFi) drug responses in RA patients has been published [16], based on the largest data obtained among machine learning studies conducted to date in RA. However, the study did not include much about feature importance analysis. In the case of AS, although machine learning to predict early TNFi users was conducted previously [17], no machine learning model has been developed to predict the responses of bDMARDs.

This study aims to examine whether machine learning can better predict the treatment responses of bDMARDs than conventional statistical methods. In addition, this study aims to identify important clinical factors that affect the treatment responses of bDMARDs through machine learning. Machine learning models including random forest (RF-method), extreme gradient boosting (XGBoost), artificial neural network (ANN), and support vector machine (SVM), are presented to predict bDMARD responses in patients with RA and AS, respectively. The prediction performances between machine learning methods, as well as with a conventional statistical method, which is logistic regression, were compared. Next, feature importance analysis was performed with the generated machine learning models to delineate the factors that are important in training models.

## Methods

### Data acquisition and participants

The data for this study were retrieved from the Korean College of Rheumatology Biologics therapy (KOBIO) registry [18], a prospective nationwide biologic therapy registry for RA, AS, and psoriatic arthritis, which includes 45 hospitals in South Korea. This registry enrolled patients who started bDMARDs with baseline clinical data and followed up annually. Our target cohort population included patients (1) with RA or AS who enrolled in the registry between December 2012 and February 2019, (2) who started bDMARDs for the first time, and (3) who were followed up for 1 year or more. All patients met the 1987 American College of Rheumatology (ACR) criteria or the 2010 ACR/European League Against Rheumatism (EULAR) criteria for RA patients, or the modified New York criteria for AS or the assessment of spondyloarthritis international society (ASAS) axial spondyloarthritis criteria for AS patients. Only bDMARD-naïve patients were included to maintain the homogeneity of the population. Patients who did not have baseline clinical data or could not check the 1-year treatment response were excluded.

The data were divided into training and independent test datasets by region of hospitals (Additional file 1: Text S1). Predictive models were generated using machine learning with only the training dataset. The independent test dataset did not participate in any training or internal validation of predictive models. It was only used for the final external validation of each trained model. Because every hospital had independent researchers and laboratory facilities and maintained individual clinical practices, we expected that dividing the test dataset by enrolled hospitals would serve similar to the independent cohort dataset. Table 1 lists the number of individuals included in each dataset.

**Table 1:** Number of training and test datasets in patients with RA and AS

	Training dataset	Independent test dataset
RA	625	322
AS	611	296

### Model design

#### Input features

The KOBIO registry collects data on demographics, comorbidities, disease activity, medication (bDMARDs, and concomitant or previous use of csDMARDs), image, extra-articular features, functional assessment, and laboratory findings as baseline clinical characteristics. We filtered input features that included only sparse information as using too many input features results in overfitting. The numbers of selected input features were 74 and 75 in RA and AS, respectively (Additional file 3: Table S1).

#### Training prediction models

Using a clinical data matrix, we trained the prediction models to distinguish between bDMARD responders and non-responders. Patients who met the ACR 20% improvement response criteria (ACR20) [19] or ASAS 20% improvement response criteria (ASAS20) [20] for RA or AS, respectively, were classified as responders. The remaining patients were classified as non-responders. ACR20 and ASAS20 have been frequently used as treatment response measures in clinical trials [21–23]. Because each input feature has a different scale, continuous features are normalized to a range of 0–1 to match the value of the categorical features, which were used directly. RF-method, XGBoost, ANN, and SVM were used to train prediction models to classify patients as responders or non-responders. In addition, a logistic regression model was constructed as a representative of a conventional statistical method to compare machine learning models. RF-method [24], XGBoost [25], and ANN [26] have several hyperparameters that must be determined before training. However, there is no consensus on the hyperparameters that are suitable for predicting clinical prognosis. Therefore, multiple machine-learning models were tested by varying the hyperparameters. The hyperparameters for RF-method include the maximum depth of a tree, total number of trees, minimum sample split, and minimum leaf samples. In the case of XGBoost, the hyperparameters include the maximum depth of a tree, learning rate, and gamma value. For the ANN, the hyperparameters include the number of hidden layers and nodes and the learning rate. The learning rate is the number of changes that newly acquired information undergoes while overriding old information, gamma refers to the minimum loss reduction required to make a further partition on a leaf node of the tree, the minimum sample split refers to the minimum number of samples required to split an internal node, and the minimum leaf samples refer to the minimum number of samples required to be at a leaf node. We chose hyperparameters with the best performance and those that performed better than logistic regression in all respects. Our training codes and generated prediction models have been made publicly accessible (https://github.com/SeulkeeLee123/KOBIO_biologics).

#### Performance evaluation

The prediction models were evaluated in three rounds of threefold cross-validation [27]. Because the responders and non-responders were unevenly distributed in the dataset, stratified cross-validation was used to divide the dataset. As mentioned earlier, only “training dataset” was used to generate prediction models. In each round, the training dataset was randomly divided into three equal sizes with stratified probability. A model was trained on two of these parts and scored on one remainder. This process was repeated thrice. Three rounds of tests resulted in a total of nine scores, and the average was used as the estimated performance score of the model. Finally, the generated models were tested with a pre-divided “independent test dataset” for external validation. The performance was measured by the accuracy, area under curve (AUC) of a receiver operating characteristic curve (ROC) and precision-recall curve, and F1 score. In addition, we used bootstrapping to calculate the confidence interval of performances [28]. A total of 1000 bootstrap iterations were used by sampling with replacement. For a confidence interval of 95%, the values at the 2.5 percentile and 97.5 percentile were selected as the lower and upper bounds, respectively.

### Feature importance analysis

Machine learning methods provide feature importance analysis, which can reveal important clinical features to predict treatment responses. For RF-method and XGBoost, the Gini importance was used for the feature importance analysis. However, compared to other machine learning methods, identifying the importance of each feature in ANN is more difficult because of its “black box” characteristics. There are several methods for evaluating feature importance despite the limitations [29, 30]. We used the differential value of the prediction score in changing each input for feature importance. In previous studies, this method was called “risk backpropagation” [30]. Furthermore, we performed analysis using clinical factors reported as important based on the feature importance analysis to evaluate whether additional clinical significance can be inferred from the results. The detailed methods for feature importance analysis are presented in the Text S1.

### Prediction models of each bDMARDs

Separate prediction models were developed for patients who use specific bDMARDs to determine the differences in models and feature importance by varying the medications. The number of medication users less than 50 were excluded from the individual analysis owing to their small size. Consequently, abatacept, adalimumab, etanercept, infliximab, and tocilizumab were chosen for RA; adalimumab, etanercept, golimumab, and infliximab were chosen for AS. An identical methodology was used to generate and evaluate the prediction models when using the entire data.

### Statistical analysis

Python (ver. 3.8.6) and R (ver. 3.6.3) [31] were used for statistical analysis. All machine learning models were generated and evaluated using the Python code. Scikit-learn (ver. 0.24.1) [32] module was used for the RF-method, SVM, and logistic regression models; xgboost (ver. 1.3.3) [25] was used for the XGBoost models. Tensorflow (2.4.1) [33] was used for the ANN models.

## Results

### Demographic and characteristics of the patients

The number of RA and AS patients included in the training dataset were 625 and 611, respectively. The demographic and baseline clinical characteristics are summarized in Tables 2 and 3, respectively. The RA and AS patients were divided into responders and non-responders, indicating those who achieved ACR20 and ASAS20 and those who did not, respectively. In the case of RA patients, responders exhibited a higher disease activity (swollen joint count [SJC] 7.00 vs. 5.87, *p* = 0.017; tender joint count [TJC] 8.78 vs. 7.68, *p* = 0.045; patient global assessment of disease activity [PtGA] 7.47 vs. 6.55, *p* < 0.001; physician global assessment of disease activity [PhGA] 6.69 vs. 6.18, *p* = 0.001; routine assessment of patient index data 3 [RAPID3] score 16.10 vs. 13.89, *p* < 0.001), lower proportion of infliximab users (40 [8.6%] vs. 24 [15.1%], *p* = 0.029), and higher proportion of tocilizumab users (165 [35.4%] vs. 29 [18.2], *p* < 0.001) than non-responders. Meanwhile, in the case of AS patients, responders exhibited a higher disease activity (Bath Ankylosing Spondylitis Disease Activity Index [BASDAI] 6.75 vs. 6.08, *p* < 0.001; BASFI 4.47 vs. 3.45, *p* < 0.001), including erythrocyte sedimentation rate (ESR, 42.46 mm/h vs. 34.82 mm/h, *p* = 0.003) and C-reactive protein (CRP) level (2.77 mg/dL vs. 2.06 mg/dL, *p* = 0.007) than non-responders. In addition, responders were younger (37.88 years vs. 40.38 years, *p* = 0.026) and taller (169.95 cm vs. 167.93 cm, *p* = 0.005).

**Table 2:** Demographics and baseline clinical characteristics of the RA patients

Baseline characteristics	Overall ***n*** = 625	Responder ***n*** = 466	Non-responder ***n*** = 159	***p***-value
Sex (male, %)	103 (16.5)	80 (17.2)	23 (14.5)	0.503
Age at baseline (years)	54.04 (12.44)	54.13 (12.40)	53.78 (12.61)	0.757
Disease duration (years)	6.78 (7.19)	6.66 (7.20)	7.14 (7.17)	0.464
Height (cm)	159.41 (7.01)	159.50 (7.08)	159.16 (6.81)	0.606
Weight (kg)	57.30 (9.72)	57.08 (9.45)	57.93 (10.47)	0.341
SJC	6.71 (5.18)	7.00 (5.10)	5.87 (5.36)	0.017
TJC	8.50 (5.98)	8.78 (5.98)	7.68 (5.93)	0.045
PtGA	7.24 (1.78)	7.47 (1.69)	6.55 (1.87)	<0.001
PhGA	6.56 (1.75)	6.69 (1.72)	6.18 (1.78)	0.001
RAPID3	15.54 (5.68)	16.10 (5.70)	13.89 (5.30)	<0.001
ESR (mm/h)	46.63 (25.66)	46.38 (25.98)	47.36 (24.77)	0.678
CRP (mg/dL)	2.34 (3.16)	2.40 (3.00)	2.16 (3.59)	0.405
HTN (%)	167 (26.7)	126 (27.0)	41 (25.8)	0.838
DM (%)	61 (9.8)	36 (7.7)	25 (15.7)	0.005
CKD (%)	5 (0.8)	3 (0.6)	2 (1.3)	0.814
Rheumatoid factor positive (%)	551 (88.2)	410 (88.0)	141 (88.7)	0.926
Anti-CCP positive (%)	494 (79.0)	373 (80.0)	121 (76.1)	0.346
Methotrexate (%)	531 (85.0)	400 (85.8)	131 (82.4)	0.357
Hydroxychloroquine (%)	175 (28.0)	136 (29.2)	39 (24.5)	0.304
Sulfasalazine (%)	85 (13.6)	67 (14.4)	18 (11.3)	0.403
Leflunomide (%)	179 (28.6)	127 (27.3)	52 (32.7)	0.226
Abatacept (%)	68 (10.9)	49 (10.5)	19 (11.9)	0.723
Adalimumab (%)	168 (26.9)	121 (26.0)	47 (29.6)	0.436
Etanercept (%)	94 (15.0)	64 (13.7)	30 (18.9)	0.151
Golimumab (%)	26 (4.2)	18 (3.9)	8 (5.0)	0.684
Infliximab (%)	64 (10.2)	40 (8.6)	24 (15.1)	0.029
Tocilizumab (%)	194 (31.0)	165 (35.4)	29 (18.2)	<0.001
Tofacitinib (%)	11 (1.8)	9 (1.9)	2 (1.3)	0.835

Data are shown in mean (standard deviation) if not otherwise specified

*SJC* swollen joint count, *TJC* tender joint count, *PtGA* patient global assessment of disease activity, *PhGA* physician global assessment of disease activity, *RAPID3* routine assessment of patient index data 3, *ESR* erythrocyte sedimentation rate, *CRP* C-reactive protein, *HTN* hypertension, *DM* diabetes mellitus, *CKD* chronic kidney disease, *anti-CCP* anti-citrullinated protein

**Table 3:** Demographics and baseline clinical characteristics of the AS patients

Baseline characteristics	Overall ***n*** = 611	Responder ***n*** = 396	Non-responder ***n*** = 215	***p***-value
Sex (male, %)	456 (74.6)	301 (76.0)	155 (72.1)	0.334
Age at baseline (years)	38.76 (13.29)	37.88 (12.85)	40.38 (13.95)	0.026
Disease duration (years)	3.56 (5.23)	3.43 (4.99)	3.80 (5.63)	0.405
Height (cm)	169.24 (8.46)	169.95 (8.37)	167.93 (8.47)	0.005
Weight (kg)	67.36 (12.65)	67.93 (12.62)	66.30 (12.67)	0.129
Peripheral arthritis (%)	234 (38.3)	155 (39.1)	79 (36.7)	0.621
Enthesitis (%)	120 (19.6)	84 (21.2)	36 (16.7)	0.222
Uveitis (%)	118 (19.3)	72 (18.2)	46 (21.4)	0.393
Dactylitis (%)	15 (2.5)	10 (2.5)	5 (2.3)	1.000
Psoriasis (%)	16 (2.6)	8 (2.0)	8 (3.7)	0.321
Inflammatory bowel disease (%)	8 (1.3)	6 (1.5)	2 (0.9)	0.814
Smoking				
Smoker + ex-smoker	179 (45.2)	284 (46.5)	105 (48.8)	0.438
Non-smoker	217 (54.8)	327 (53.5)	110 (51.2)	
BASDAI	6.52 (1.70)	6.75 (1.61)	6.08 (1.77)	<0.001
BASFI	4.11 (2.53)	4.47 (2.47)	3.45 (2.51)	<0.001
ESR (mm/h)	39.77 (30.89)	42.46 (31.26)	34.82 (29.64)	0.003
CRP (mg/dL)	2.52 (3.12)	2.77 (3.32)	2.06 (2.64)	0.007
HTN (%)	96 (15.7)	48 (12.1)	48 (22.3)	0.001
DM (%)	18 (2.9)	11 (2.8)	7 (3.3)	0.934
CKD (%)	0 (0.0)	0 (0.0)	0 (0.0)	NA
HLA-B27 positive (%)	543 (88.9)	358 (90.4)	185 (86.0)	0.133
NSAIDs use (%)	521 (85.3)	334 (84.3)	187 (87.0)	0.449
Methotrexate (%)	44 (7.2)	23 (5.8)	21 (9.8)	0.100
Sulfasalazine (%)	35 (5.7)	19 (4.8)	16 (7.4)	0.246
Adalimumab (%)	253 (41.4)	165 (41.7)	88 (40.9)	0.928
Etanercept (%)	74 (12.1)	47 (11.9)	27 (12.6)	0.905
Golimumab (%)	115 (18.8)	81 (20.5)	34 (15.8)	0.196
Infliximab (%)	169 (27.7)	103 (26.0)	66 (30.7)	0.253

Data are shown in mean (standard deviation) if not otherwise specified.

*BASDAI* Bath Ankylosing Spondylitis Disease Activity Index, *BASFI* Bath Ankylosing Spondylitis Functional Index, *ESR* erythrocyte sedimentation rate, *CRP* C-reactive protein, *HTN* hypertension, *DM* diabetes mellitus, *CKD* chronic kidney disease, *HLA* human leukocyte antigen, *NSAIDs* non-steroidal anti-inflammatory drugs

### Prediction model optimization

**Fig. 1:**
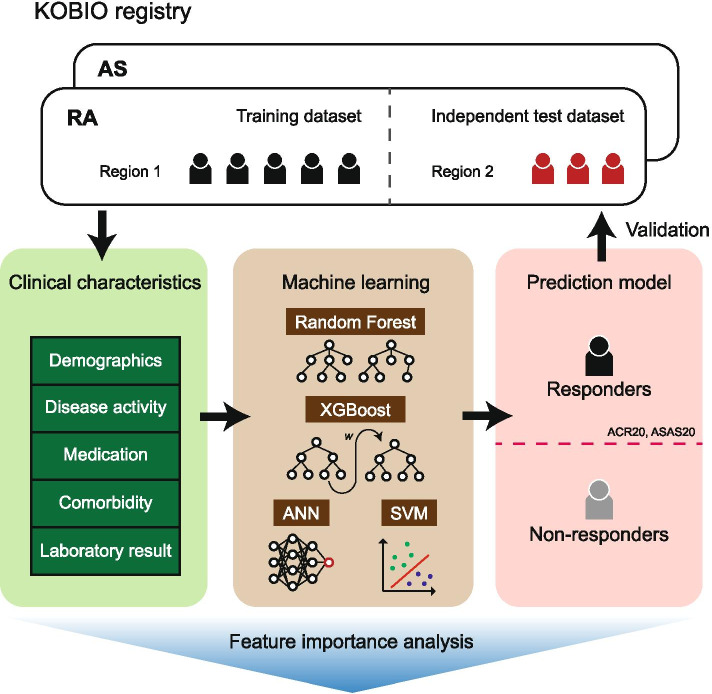
Overview of the prediction model for responses of bDMARDs. The model uses data on baseline clinical characteristics, including comorbidities, disease activity, medication, image, extra-articular features, functional assessment, and laboratory findings, to classify the patient as a responder or non-responder

Prediction models that classified patients as responders or non-responders were trained using RF-method, XGBoost, ANN, SVM, and logistic regression (Fig. 1). The RF-method, XGBoost, and ANN models were significantly different in terms of their hyperparameters. Thus, we trained them repeatedly to determine an appropriate hyperparameter set for the input dataset (Additional file 2: Figures S1-6). Hyperparameters of better performing models than the logistic regression model were selected in terms of all four performance measures (accuracy, AUC of ROC curve, F1 score, and AUC of precision-recall curve). The chosen hyperparameter sets of each model are listed in Additional file 3: Table S2.

### Performance of predicting bDMARDs responses

**Fig. 2:**
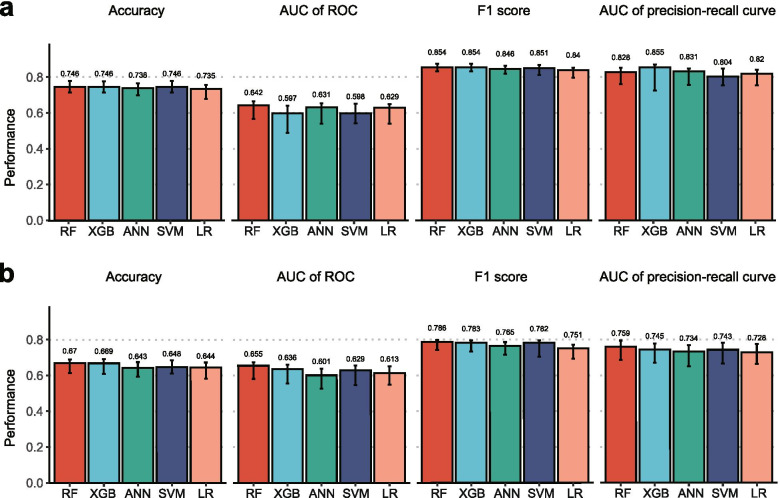
Performance of models trained using various methods (RF-method, XGBoost, ANN, SVM, and logistic regression) with training dataset, in terms of accuracy, AUC of the ROC curve, F1 score, and AUC of the precision-recall curve. **a** RA patients. **b** AS patients. LR, logistic regression

Performance of various models was compared in terms of the accuracy, AUC of the ROC and precision-recall curves, and F1 score. The prediction models were evaluated in three rounds of three-fold cross-validation. In both disease cohorts, RF-method showed the best performance among the various methods in almost all fields (Fig. 2). However, the differences were within the confidence intervals calculated using bootstrap methods. Prediction models with RA patients exhibited better performance in general. Although the different structures of the training dataset could affect the performance of the prediction methods, it is likely that the performance differs in reality because all four measures were better in RA patients.

### Evaluation on independent test dataset

**Fig. 3:**
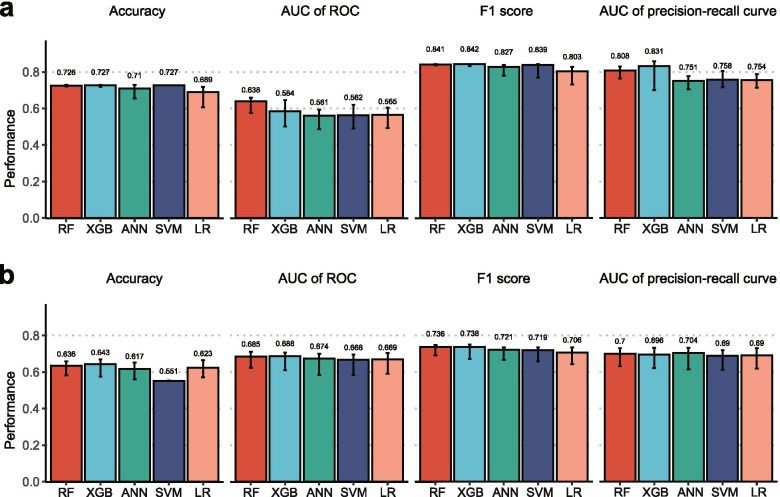
Performances of models trained using various methods (RF-method, XGBoost, ANN, SVM, and logistic regression) with independent test dataset, in terms of accuracy, AUC of the ROC curve, F1 score, and AUC of the precision-recall curve. **a** RA patients. **b** AS patients. LR, logistic regression

To validate the performance of the prediction model, we excluded data on specific hospitals from the processes to formulate prediction models and the excluded data were used as an independent test dataset. The performance of previously obtained prediction models was evaluated with data from an independent test dataset. In the case of RA patients, the prediction performances of the RF-method and XGBoost models were higher than those of the logistic regression model (Fig. 3a). RF-method and XGBoost showed similar performances in all four performance measures; however, RF-method exhibited more robust results with the bootstrap method. The RF-method model showed better prediction performance than the logistic regression model, even considering the 95% confidence interval calculated using bootstrap methods (accuracy 0.726 [95% confidence interval (CI) 0.725–0.730] vs. 0.689 [0.606–0.717], AUC of the ROC 0.638 [0.576–0.658] vs. 0.565 [0.493–0.605], F1 score 0.841 [0.837–0.843] vs. 0.803 [0.732–0.828], AUC of the precision-recall curve 0.808 [0.763–0.829] vs. 0.754 [0.714–0.789]). The ANN and SVM did not show superior prediction performance. In contrast with RA patients, prediction performances between the machine learning methods and logistic regression in AS patients did not significantly differ (Fig. 3b).

### Feature importance analysis

**Fig. 4:**
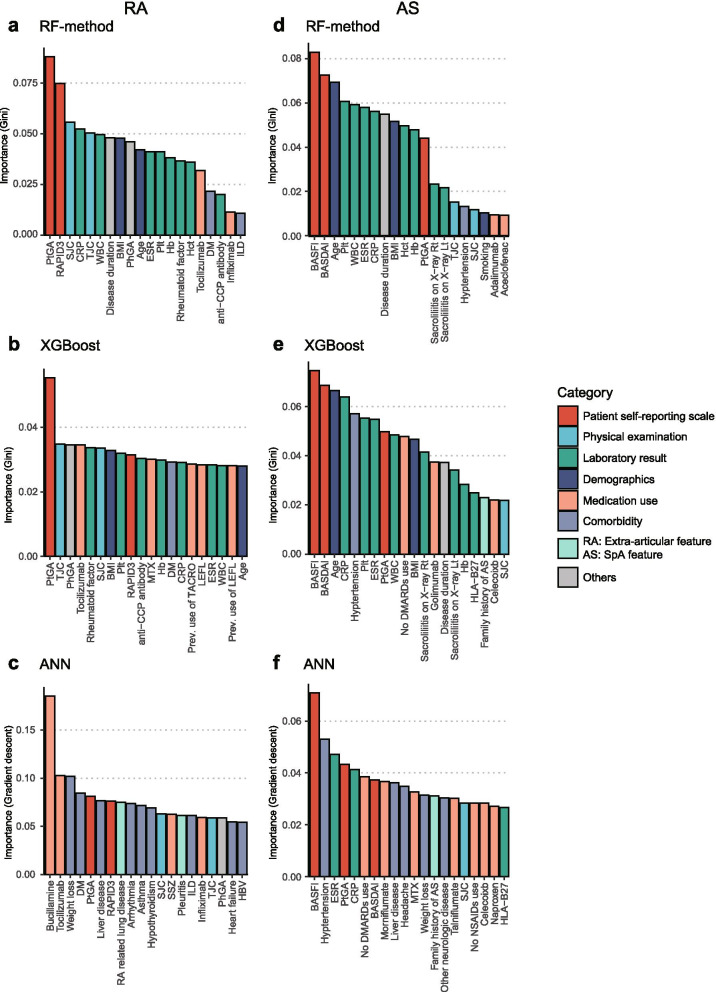
Result of feature importance analysis from the best performing models of each machine learning method. The *X*-axis represents the input clinical features. The Y-axis represents the feature importance score calculated using the Gini importance or risk backpropagation methods in RF-method/XGBoost and ANN, respectively. The color of columns represents the categories in which the feature was included. Top 20 important features are shown in figures. Feature importance of **a** RF-method model, **b** XGBoost model, and **c** ANN model in patients with RA. Feature importance of **d** RF-method model, **e** XGBoost model, and **f** ANN model in patients with AS. WBC, white cell count; BMI, body mass index; Plt, platelet; Hb, hemoglobin; Hct, hematocrit; DM, diabetes mellitus; anti-CCP, anti-cyclic citrullinated protein; ILD, interstitial lung disease; MTX, methotrexate; TACRO, tacrolimus; LEFL, leflunomide; SSZ, sulfasalazine

Feature importance analysis was implemented using the best-performing models of each RF-method, XGBoost, and ANN methods. Gini importance method was used for RF-method and XGBoost models, and risk backpropagation method was used for ANN models to calculate feature importance. The top three important input features of the RF-method model in RA patients were PtGA, RAPID3, and SJC (Fig. 4). Except for the ANN model in RA patients, the most important input feature was PtGA, which is a self-reported scale, rather than more objective features, such as laboratory results or physical examination. Among the three machine learning methods, ANN exhibited the worst prediction performance. In the feature importance analysis, the ANN showed significantly different results from the other two methods. Considering the prediction performance of the ANN model, the feature importance results of the ANN model were regarded as unreliable compared to other methods. In the case of AS, the most important input feature of all three machine learning methods was BASFI, which is a self-reported functional assessment score for AS (Fig. 4), followed by BASDAI in the RF-method and XGBoost models. By combining self-reported scales predicted to be important, we attempted to analyze whether additional information could be found through a conventional statistical method; however, the results were inconsistent (Text S1).

### Prediction models of different bDMARDs

The training dataset was divided based on the type of bDMARD. RA patients were divided into abatacept, adalimumab, etanercept, infliximab, and tocilizumab users; AS patients were divided into adalimumab, etanercept, golimumab, and infliximab users. The performance of the RF-method and logistic regression models in RA patients did not differ from each other in all medication users (Additional file 2: Figure S7). The feature importance analysis results of the prediction models did not show consistent results for each medication cohort in the RA patients (Additional file 2: Figure S8). This could be because of the small size of the dataset of individual medication users. The performance of the RF-method models in AS patients showed similar results (Additional file 2: Figure S9). However, the RF-method prediction model in adalimumab users in AS patients showed better performance than the logistic regression model, particularly when the model was tested using an independent dataset, despite the borderline differences. Feature importance analysis of the RF-method model of adalimumab users in AS patients showed that the most important input feature was BASFI, followed by BASDAI (Additional file 2: Figure S10). This result was similar to that of the entire AS dataset. The number of patients in the adalimumab cohort with AS was 253, the largest among the individual medication datasets. The size of the training dataset could be the reason for the better performance of the prediction model in the adalimumab cohort. In general, we could not formulate a prediction model for individual medication use with reasonable performance in most cases, and the primary reason seemed to be the size of the cohort.

## Discussion

Various machine learning models were presented to classify the treatment responses of bDMARDs in RA and AS patients. In RA patients, RF-method was the most suitable method to predict treatment responses more accurately than the conventional statistical method, which is logistic regression. However, machine learning models to predict treatment responses of biologic agents in AS patients are not superior in contrast to RA. According to the feature importance analysis, patient self-reporting scales were the most important input features in both diseases. Only a few previous studies have been published to predict treatment responses to biologic agents in RA patients [16, 34]. However, the present study includes a more detailed feature importance analysis than previous studies. Furthermore, this is the first attempt to predict the treatment responses of bDMARDs in AS patients.

We implemented various machine learning methods to predict treatment responses, including RF-method, XGBoost, ANN, and SVM. Both RF-method and XGBoost are ensemble models that consist of numerous small decision trees. RF-method is based on a bagging algorithm, and XGBoost is based on a gradient boosting algorithm. Although SVM is relatively older, it exhibits a satisfactory performance in simple image classification with little computational burden. ANNs are gradually gaining popularity as they obtain successful results in various fields, such as image classification. However, decision-tree-based algorithms show better performance in certain circumstances, such as small, tabular data [35]. RF-method showed better prediction performance than ANN in RA patients in this study. In addition, the optimal ANN prediction model had only one (RA) or two (AS) hidden layers, which are too shallow to obtain the advantage of ANN. Therefore, our input data seemed unsuitable for the ANN. This could be because of the relatively small size of the input data.

RF-method showed better prediction performance than logistic regression in patients with RA but not in those with AS. In addition, the prediction performance of the various models was lower in AS patients. Determining the exact reason requires further research and is beyond the scope of this study, although some speculation can be made. The number of data points was slightly smaller in AS; however, the difference was only 5–10% of all patients. The number of input features of the AS was higher than that of the RA. RA had a more unbalanced responder/non-responder proportion, which generally had a negative effect on machine learning results. Thus, the differences in the prediction performance were unlikely because of the structure of the input dataset. If so, we could assume that the input features were insufficient to predict the treatment response of bDMARDs in patients with AS. Heritability analysis implied that AS has more genetic factors than RA, with higher heritability of approximately 80–90% [36–39] vs. 50–60% [40, 41] in AS and RA, respectively. Previous studies have shown that genetic features could affect the response of bDMARDs in patients with AS [42, 43]. In addition, there have been pilot studies of transcriptome analysis [44, 45] to predict the responses of bDMARDs in patients with AS. Therefore, multi-omics data, including genetics and transcriptomics, may improve prediction performance.

Feature importance analysis can provide insights into clinical factors. In this study, machine learning models revealed that the patient self-reporting scales, PtGA and BASFI in RA and AS patients, respectively, were the most important factors for predicting treatment responses. It is quite surprising because they are more important than more objective clinical features, such as laboratory results (ESR and CRP) and physical examination (SJC and TJC). Previous studies reported patient self-reporting scales, such as RAPID3 [46] or BASFI [47] as predictors of bDMARD treatment. However, their relative importance compared with other objective disease activities or functional measures has not been studied. In addition, given that the results of feature importance were similar except for ANN in RA patients, which had inferior performances, the result of the feature important analysis was robust.

The prediction models were trained for each medication use separately. However, the performance of prediction models using RF-method was not superior to that of logistic regression models in each medication dataset. Only the prediction model of adalimumab users in patients with AS using RF-method had a borderline superior result to the logistic regression model. The results of the feature importance analysis for each medication user were not consistent. Again, only the model of adalimumab patients in patients with AS showed similar results to the entire cohort in the feature importance analysis. Adalimumab users in patients with AS occupied the largest patient group with 253 individuals, while the other cohorts comprised less than 200 patients. Therefore, the size of the patient group must be an important factor in generating a proper predictive model, and approximately 250 people could be the lower limit of size.

However, our approach had some limitations. First, even though we divided part of the dataset by the region of hospitals as an independent test dataset and did not participate in any part of the training machine learning model, the validation cohort was not retrieved from a completely different cohort. However, forty-five hospitals were involved in the KOBIO cohorts, and each hospital had an independent enrollment process, assessment physician, and laboratory institution. Thus, we expect that pre-divided test dataset represents an independent cohort. Second, all participants were Koreans, therefore we do not assure that the models we generated showed similar results in other populations. When applied to other populations, new patient data or feature selection may be required in advance.

## Conclusions

In conclusion, we developed several machine learning models that could predict the treatment responses of biologic agents in patients with RA and AS. The best-performing model was trained using RF-method in patients with RA. The model performs better than the conventional statistical method, logistic regression. Given the input clinical features, machine learning models have no advantages compared to a logistic regression model in patients with AS. Feature importance analysis shows that patient self-reporting scales, PtGA and BASFI in RA and AS patients, respectively, are the most important input features for machine learning prediction models.

## Supplementary Information

**Additional file 1: Text S1.** Detailed methods for dividing test dataset and feature importance analysis, and additional analysis with features reported from the feature importance analysis.

**Additional file 2: Figure S1-3.** Prediction performance of machine learning methods by varying hyperparameters in RA patients. **Figure S4-6.** Prediction performance of machine learning methods by varying hyperparameters in AS patients. **Figure S7.** Performance of RF-method and logistic regression models in RA patients divided by the type of bDMARDs. **Figure S8.** Result of the feature importance analysis of the best-performing RF-method model in RA patients divided by the type of bDMARDs. **Figure S9.** Performance of RF-method and logistic regression models in AS patients divided by the type of bDMARDs. **Figure S10.** Feature importance analysis of the best-performing RF-method model in AS patients divided by the type of bDMARDs. **Figure S11.** Correlation analysis between input features that reported the feature importance analysis. **Figure S12.** Linear regression analysis with dot plot results for input features. **Figure S13.** Prediction performances of logistic regression models using patient self-reported scales and their combinations.

**Additional file 3: Table S1.** List of input features. **Table S2.** Chosen hyperparameter sets for each model.

## Data Availability

All data generated or analyzed during this study are included in this published article. Our training codes and generated prediction models have been made publicly accessible (https://github.com/SeulkeeLee123/KOBIO_biologics).

## Abbreviations

ACR: American College of Rheumatology
ACR20: ACR 20% improvement response criteria
ANN: Artificial neural network
AS: Ankylosing spondylitis
ASAS: Assessment of Spondyloarthritis International Society
ASAS20: ASAS 20% improvement response criteria
AUC: Area under curve
BASDAI: Bath Ankylosing Spondylitis Disease Activity Index
BASFI: Bath Ankylosing Spondylitis Functional Index
bDMARDs: Biologic disease-modifying antirheumatic drugs
CI: Confidence interval
CRP: C-reactive protein
csDMARDs: Vonventional synthetic disease-modifying rheumatic drugs
ESR: Erythrocyte sedimentation rate
EULAR: European League Against Rheumatism
KOBIO: Korean College of Rheumatology Biologics and Targeted Therapy
PhGA: Physician global assessment of disease activity
PtGA: Patient global assessment of disease activity
RA: Rheumatoid arthritis
RAPID3: Routine assessment of patient index data 3
ROC: Receiver operating characteristic curve
SJC: Swollen joint count
SVM: Support vector machine
TJC: Tender joint count
XGBoost: Extreme gradient boosting
