# Supplementary material for: Machine learning-based prediction model for responses of bDMARDs in patients with rheumatoid arthritis and ankylosing spondylitis
Source: Arthritis Res Ther. 2021 Oct 9;23:254. doi: 10.1186/s13075-021-02635-3 (PMC8501710; doi:10.1186/s13075-021-02635-3)
Supplement: Supplementary file 1 — Additional file 1: Text S1. Detailed methods for dividing test dataset and feature importance analysis, and additional analysis with features reported from the feature importance analysis. [file 13075_2021_2635_MOESM1_ESM.docx]

**Machine learning-based prediction model for responses of bDMARDs in patients with rheumatoid arthritis and ankylosing spondylitis**

Seulkee Lee^1^, Seonyoung Kang^1^, Yeonghee Eun^1^, Hong-Hee Won^2^, Hyungjin Kim^1^, Jaejoon Lee^1^, Eun-Mi Koh^1^, and Hoon-Suk Cha^1^ *

^1^ Department of Medicine, Samsung Medical Center, Sungkyunkwan University School of Medicine, Seoul, Republic of Korea

^2^ Samsung Advanced Institute for Health Sciences & Technology (SAIHST), Sungkyunkwan University, Samsung Medical Center, Seoul, Republic of Korea

**Text S1**

**Dividing test dataset**

We divided the entire cohort into training and test datasets based on the region of the hospitals. Because all hospitals have their own enrolling processes and environments, we could obtain the most independent test dataset by dividing by hospital. The registry had 13 codes to represent the region of hospitals. Table 1 lists the number of patients in each region and the dataset for each region.

**Table 1.** Number of patients and the type of dataset belonging to each region.

| **Region** | **RA** | | **AS** | |
| --- | --- | --- | --- | --- |
|  | **Number of patients** | **Type of dataset** | **Number of patients** | **Type of dataset** |
| Seoul 1 | 256 | Test dataset | 363 | Training dataset |
| Seoul 2 | 229 | Training dataset | 117 | Test dataset |
| Incheon | 21 | Training dataset | 40 | Test dataset |
| Gyeonggi-do | 53 | Training dataset | 100 | Training dataset |
| Chungcheong-do | 66 | Test dataset | 84 | Test dataset |
| Gangwon-do | 0 | N/A | 6 | Training dataset |
| Cheonan | 14 | Training dataset | 1 | Training dataset |
| Gyeongsangbuk-do | 123 | Training dataset | 45 | Test dataset |
| Busan | 4 | Training dataset | 15 | Training dataset |
| Gyeongsangnam-do | 17 | Training dataset | 10 | Test dataset |
| Jeollanam-do | 30 | Training dataset | 62 | Training dataset |
| Jeollabuk-do | 130 | Training dataset | 62 | Training dataset |
| Jeju | 4 | Training dataset | 2 | Training dataset |

**Feature importance analysis**

**RF and XGBoost.** Feature importance analysis methods of RF and XGBoost are based on the Gini impurity by default from suppliers. The Gini impurity is the probability of incorrectly classifying a randomly chosen element in the dataset, if it were randomly labeled according to the class distribution in the dataset.

$$Gini= \sum_{i=1}^{C} p\left( i \right)\times(1-p\left( i \right))$$

where *C* and $p\left( i \right)$ represent the number of classes and the probability of randomly picking an element of class *i*, respectively*.*

For training a decision tree, the best split is determined by maximizing the Gini gain, which is calculated by subtracting the weighted impurities of the branches from the original impurity.

**ANN.** We used the differential value of the prediction score to change each input variable for feature importance. In a previous study, this method was called ‘risk backpropagation’ [1].

$$Feature importance_{i}= \frac{\partial prediction score}{\partial input_{i}}$$

where $input_{i}$ = (value of *i*-th variable).

The more important the role an input variable plays in model training, the more the output value (in this case, the prediction score) will change as the input changes; that is, when the output value is differentiated into a specific input variable, the larger the value of the differential, the more important the variable is in training the prediction model. We calculated the differential value of each input variable to reveal the feature importance.

**Additional analysis with features reported from feature importance analysis**

As reported in our main analysis, patient self-reporting scales play an important role in feature importance analysis. Thus, we checked whether additional clinical significance can be derived by employing patient self-reporting scales without using machine learning. Accordingly, we examine the relationship between patient self-reporting scales and other high-ranked features in the feature importance analysis. In addition, the relationship between treatment responsiveness and the important features, including patients self-reporting scales, was demonstrated through univariate regression analysis and predictive performance. In this additional analysis, correlation analyses between variables were performed using the training dataset to determine the data structure of the predictive model. Conversely, the performance of predictive models was analyzed in the independent test dataset because using such a test dataset provides an effective validation of the performance.

**Correlation analysis between important input variables.** We checked the correlation between features that reported high ranks in the feature importance analysis of the predictive model using random forest (RF-method) for each disease. We used the top 12 ranked features in further analysis because the lowest rank of patient self-reporting scale in ankylosing spondylitis (AS), patient global assessment of disease activity (PtGA), was 12th. Table 2 lists the features employed for the correlation analysis.

Table 2 Features used for correlation analysis. The features were retrieved from the top 12 ranked features in the feature importance analysis of the predictive model using the RF-method.

| Rank* | RA | | AS | |
| --- | --- | --- | --- | --- |
|  | Feature | *P*-value^†^ | Feature | *P*-value^†^ |
| 1st | PtGA | <0.001 | BASFI | <0.001 |
| 2nd | RAPID3 | <0.001 | BASDAI | <0.001 |
| 3rd | SJC | 0.018 | Age | 0.027 |
| 4th | CRP | 0.407 | Platelet | 0.105 |
| 5th | TJC | 0.046 | WBC | 0.387 |
| 6th | WBC | 0.691 | ESR | 0.004 |
| 7th | Disease duration | 0.463 | CRP | 0.008 |
| 8th | BMI | 0.183 | Disease duration | 0.405 |
| 9th | PhGA | 0.002 | BMI | 0.961 |
| 10th | Age | 0.757 | Hct | 0.765 |
| 11th | ESR | 0.678 | Hb | 0.599 |
| 12th | Platelet | 0.506 | PtGA | <0.001 |
| * The rank from the feature importance analysis using the RF-method model to predict treatment response of tumor necrosis factor-alpha inhibitors in each disease.  ^†^ *P*-values of univariate logistic regression analysis between each feature and treatment response of biologic disease-modifying antirheumatic drugs.  *RA* rheumatoid arthritis, *AS* ankylosing spondylitis, PtGA patient global assessment of disease activity, *BASFI* Bath Ankylosing Spondylitis Functional Index, *RAPID3* routine assessment of patient index data 3, *BASDAI* Bath Ankylosing Spondylitis Disease Activity Index, *SJC* swollen joint count, *CRP* C-reactive protein, *TJC* tender joint count, *WBC* white blood cell, *ESR* erythrocyte sedimentation rate, *BMI* body mass index, *PhGA* physician global assessment of disease activity, *Hct* hematocrit, *Hb* hemoglobin. | | | | |

As expected, correlation analysis showed a correlation within each group of patient self-reporting scales (PtGA and routine assessment of patient index data 3 (RAPID3) for rheumatoid arthritis (RA), and Bath Ankylosing Spondylitis Functional Index (BASFI), Bath Ankylosing Spondylitis Disease Activity Index (BASDAI), and PtGA for AS); physical examination (swollen joint count (SJC) and tender joint count (TJC) for RA); and blood test results (erythrocyte sediment rate (ESR), C-reactive protein (CRP), white blood cell (WBC) count, platelet count for RA, hemoglobin level, hematocrit, platelet level, ESR, and CRP for AS; Figure S11). However, patient demographics (age and body mass index) and disease duration showed a relatively small correlation with other features. Regarding the patient self-reporting scales, the correlations between the features within the patient self-reporting scale group were greater than those outside the group.

In addition, we determined whether the relationship between each input feature was linear (Figure S12). We observed a nonlinear relationship between the patient self-reporting scale and the other variables. In addition, even within the patient self-reporting scale group, it was difficult to determine a linear relationship because all R-squared values of the linear regression analysis were ​​less than 0.3.

**Relationship between each feature and treatment response.** Univariate logistic regression analysis was performed to determine the correlation between each feature and the treatment response of biologic disease-modifying antirheumatic drugs (bDMARDs). Patient self-reporting scales were related to treatment response in the regression analysis results, with a statistical significance in both diseases (Table 2). A considerable number of high-ranked features based on the feature importance analysis were also associated with the treatment response in logistic regression analysis. However, the order of ranks in the feature importance analysis did not match the order of significance in the logistic regression analysis. Therefore, the results of the feature importance analysis were difficult to infer only from the results of conventional statistical analysis.

**Prediction model using a combination of patient self-reporting scales** We analyzed whether the combination of the patient self-reporting scales improved the predictive ability of the treatment response, compared to individual use. Because we have determined the combination of features through machine learning, the key to this analysis is to analyze the change in the predictive ability when combining features through logistic regression analysis, which is a conventional statistical method. Responders and non-responders were separated according to the same criteria as the main analysis. The prediction model was generated using only the training dataset, and the performance was evaluated using an independent test dataset. Datasets were divided into training and independent test datasets according to the region of hospitals, as in the main analysis. The input features were the patient self-reporting scales of each disease: PtGA and RAPID3 for RA; BASDAI, BASFI, and PtGA for AS. We generated a logistic regression model using individual input features and a combination of features.

The performance of each model was measured by the area under the curve (AUC) of the receiver operating characteristic (ROC) curve and precision-recall curve. The prediction models were evaluated in three rounds of three-fold cross-validation. In patients with RA, the AUCs of the ROC curve of each model were 0.594, 0.608, and 0.609, and the AUCs of the precision-recall curve of each model were 0.776, 0.774, and 0.780 with the input of PtGA, RAPID3, and the combination of the two variables, respectively (Figure S13A). When the two variables were combined, the performance of the prediction model was better than when each variable was used separately; however, the difference was negligible. Thus, the combination of patient self-reporting scales did not play a significant role in the prediction of treatment responses using logistic regression. Moreover, these results were even better than those of the logistic regression model using all combining baseline input features (AUC of the ROC: 0.565, AUC of the precision-recall curve: 0.754). However, all three models showed inferior performances, compared to the prediction model generated by the RF-method (AUC of the ROC: 0.638, AUC of the precision-recall curve: 0.808). Therefore, predictive performance is reduced when variables that have moderate effect on treatment response are included as input variables of logistic regression with a dataset of RA patients. This phenomenon probably results from overfitting, and machine learning can likely overcome this problem.

In patients with AS, the AUCs of the ROC curve of each model were 0.573, 0.633, 0.578, and 0.620, and those of the precision-recall curve of each model were 0.584, 0.625, 0.575, and 0.612 with the input of BASDAI, BASFI, PtGA, and the combination of the three variables, respectively (Figure S13B). When the three variables were combined, the performance of the prediction model was better than that of the model generated by BASDAI and PtGA, but similar to the model using BASFI. Therefore, the combination of patient self-reporting scales showed inconsistent results compared with the use of each variable separately. Unlike the dataset of AS, the performance was lower than that of the logistic regression model using all combining baseline input features (AUC of the ROC: 0.669, AUC of the precision-recall curve: 0.690). As mentioned in the Results section, the performance of the prediction model generated using the RF-method (AUC of the ROC: 0.685, AUC of the precision-recall curve: 0.700) was not significantly superior to the logistic regression model in patients with AS. Therefore, the amount of input information is more important than overfitting in the AS dataset. Although machine learning could not solve the lack of input information, it can address overfitting issues better than conventional statistical models, which may be a reason why machine learning had more significant performance improvement in patients with RA than AS.

**Reference**

1. Yousefi S, Amrollahi F, Amgad M, Dong C, Lewis JE, Song C, et al. Predicting clinical outcomes from large scale cancer genomic profiles with deep survival models. Sci Rep. 2017;7(1):11707.
