# Supplementary material for: Machine learning-based prediction model for responses of bDMARDs in patients with rheumatoid arthritis and ankylosing spondylitis
Source: Arthritis Res Ther. 2021 Oct 9;23:254. doi: 10.1186/s13075-021-02635-3 (PMC8501710; doi:10.1186/s13075-021-02635-3)

Figure S1. Prediction performances of RF-method model by varying maximum depths of a tree, numbers of trees, minimum sample split, and minimum leaf samples in patients with RA. The X-axis represents the differing maximum depths of a decision tree. The Y-axis represents the (a) accuracy, (b) AUC of the ROC curve, (c) F1 score, and (d) AUC of the precision-recall curve. The performances of the models based on the numbers of trees are depicted as a line. The dashed red horizontal lines represent the prediction performance of logistic regression models.

(a)

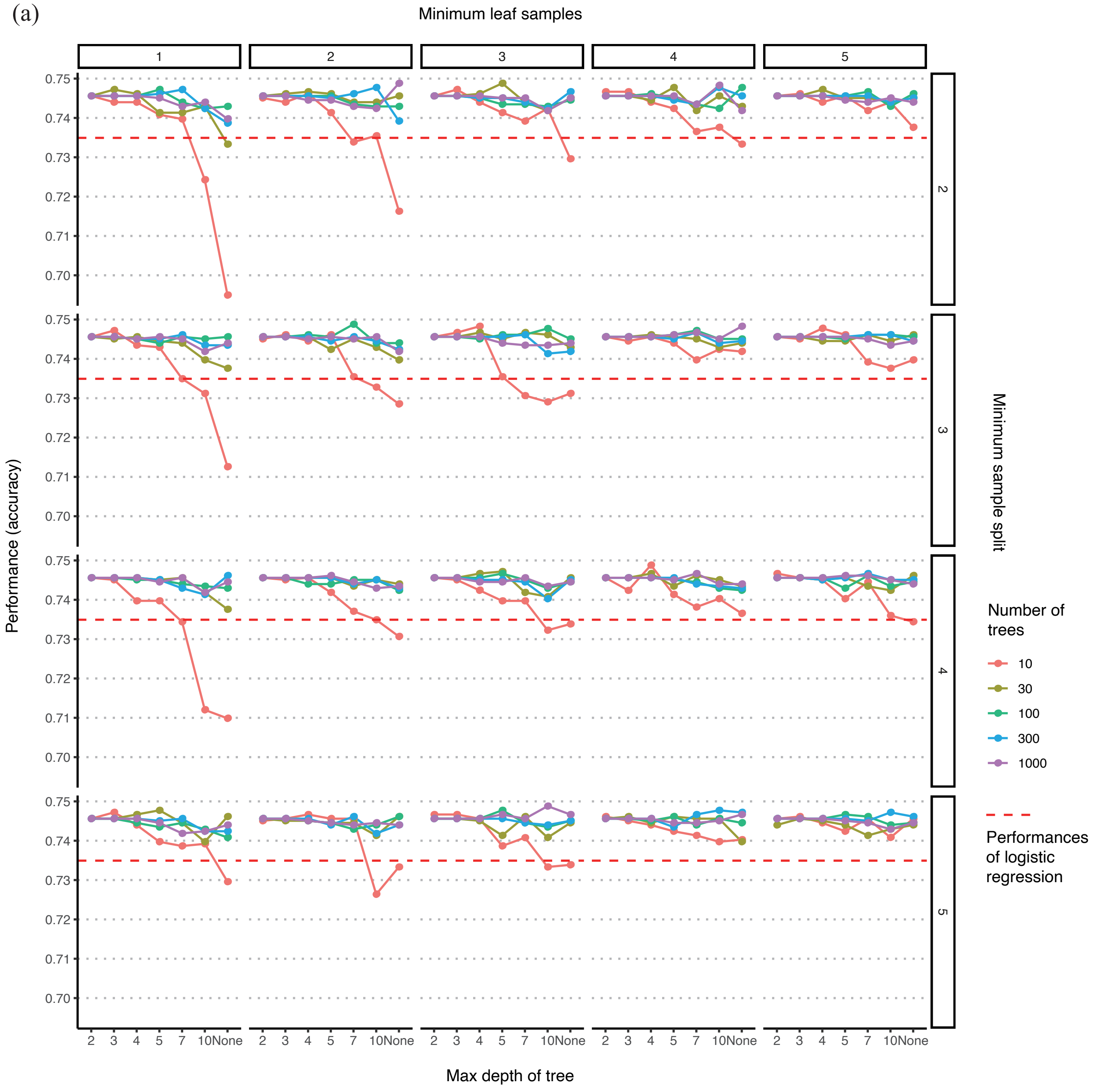

(a)

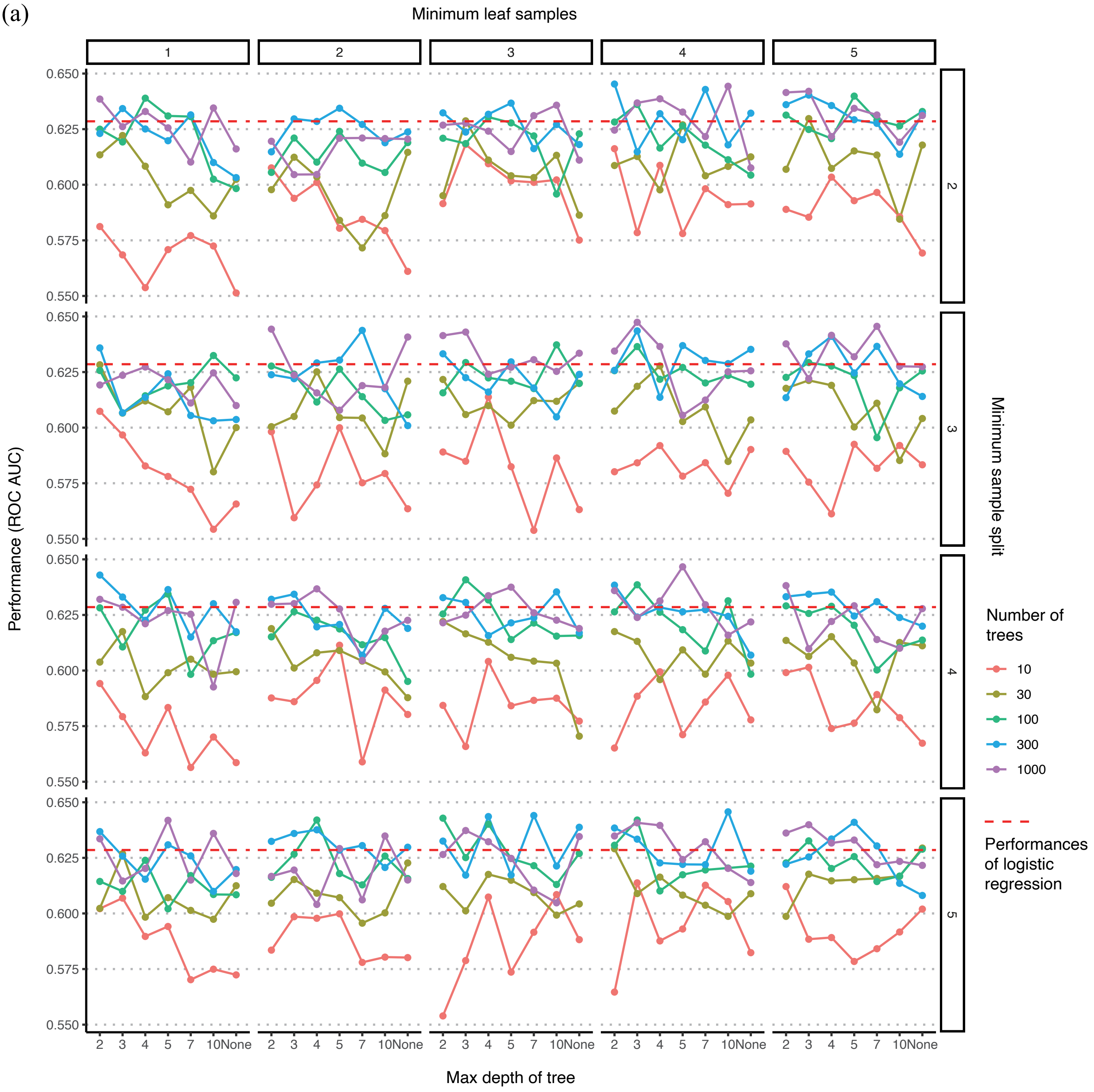
Minimum leaf samples
1
2
3
4
5
Minimum sample split
2
3
4
5
Performance (ROC AUC)
0.650
0.625
0.600
0.575
0.550
Max depth of tree
2
3
4
5
7
10
None
Number of trees
10
30
100
300
1000
Performances of logistic regression

(c)

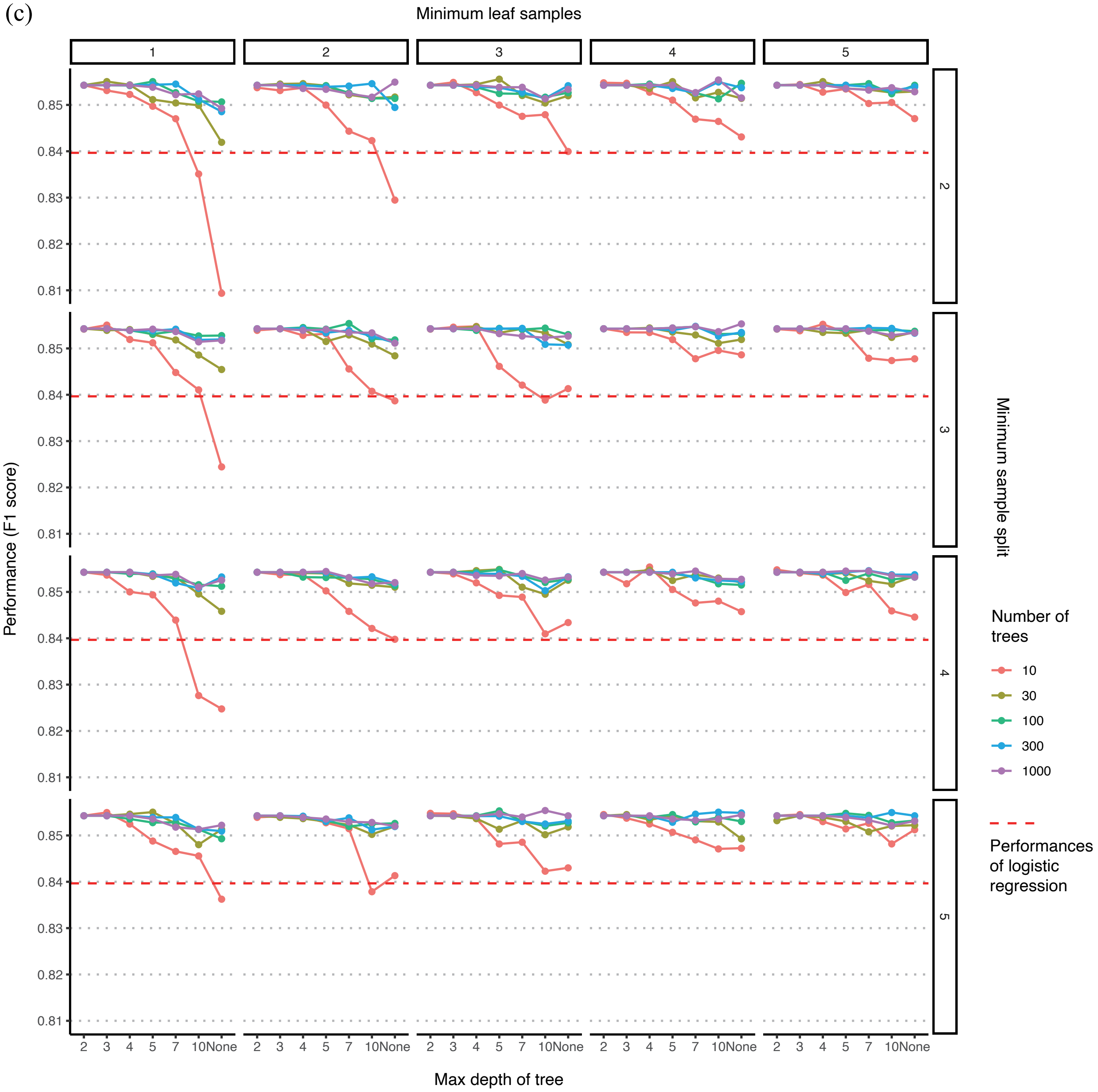
Minimum leaf samples
1
2
3
4
5
Performance (F1 score)
0.85
0.84
0.83
0.82
0.81
Minimum sample split
2
3
4
5
Max depth of tree
2 3 4 5 7 10None
Number of trees
10
30
100
300
1000
Performances of logistic regression

(d)

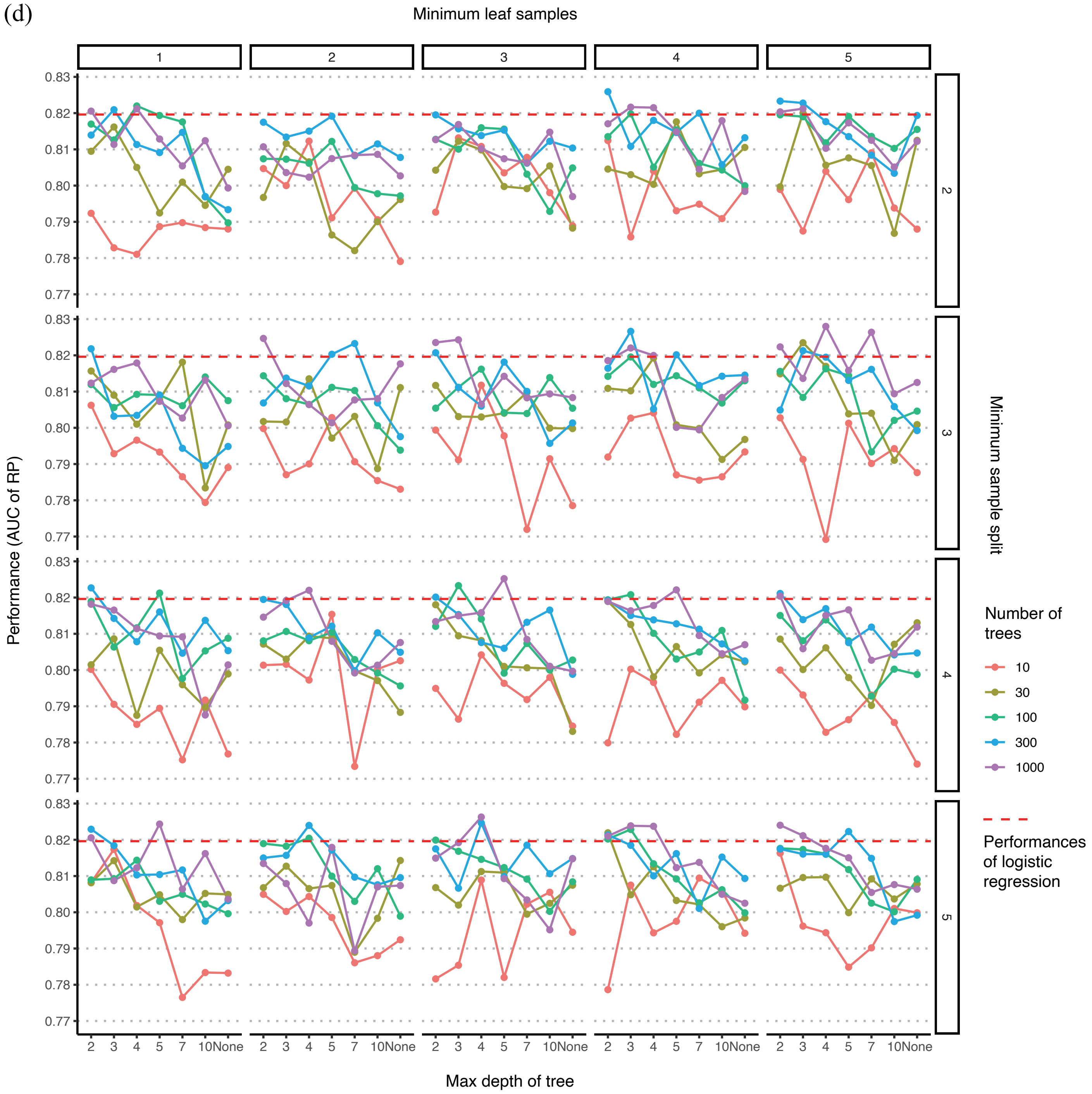

Minimum leaf samples
1
2
3
4
5
Minimum sample split
2
3
4
5
Performance (AUC of RP)
0.83
0.82
0.81
0.80
0.79
0.78
0.77
Max depth of tree
2 3 4 5 7 10None
Number of trees
10
30
100
300
1000
Performances of logistic regression

Figure S2. Prediction performances of XGBoost model by varying maximum depths of a tree and numbers of trees in patients with RA. The X-axis represents the differing maximum depths of a decision tree. The Y-axis represents the (a) accuracy, (b) AUC of the ROC curve, (c) F1 score, and (d) AUC of the precision-recall curve. The performances of the models based on the numbers of trees are depicted as a line. The dashed red horizontal lines represent the prediction performance of logistic regression models.

(a)

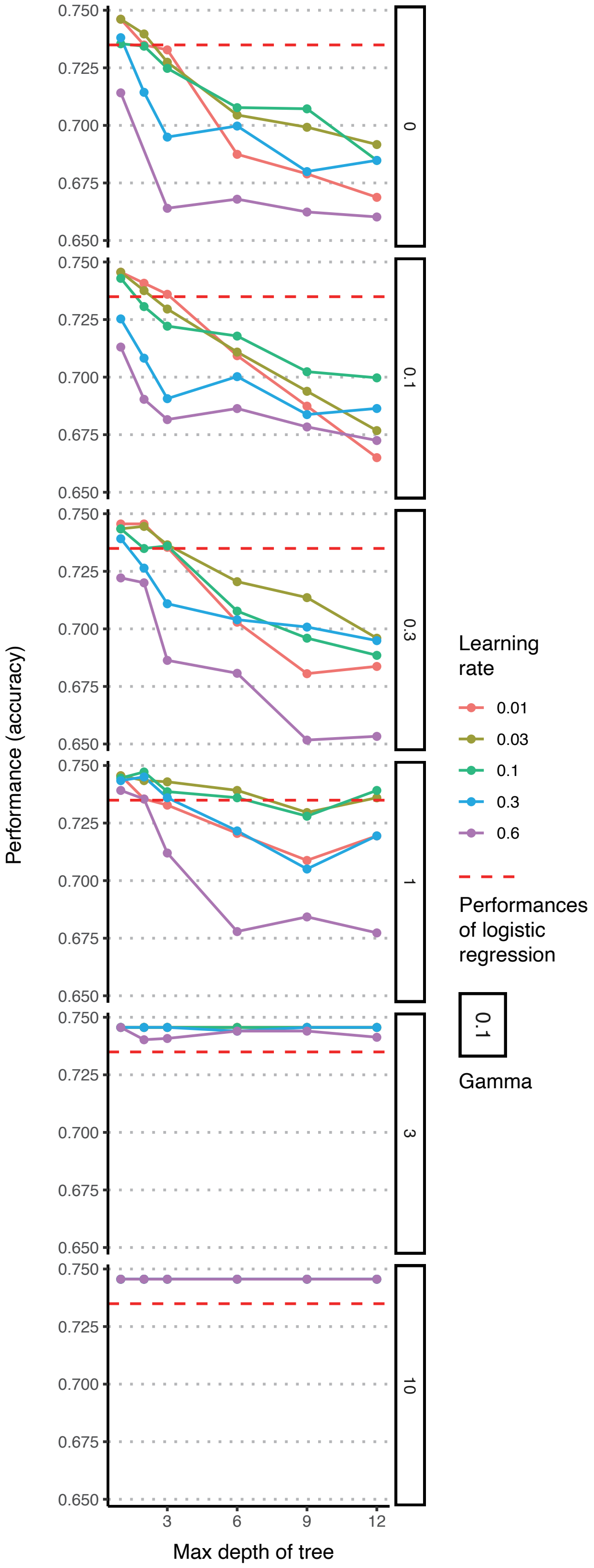

(b)

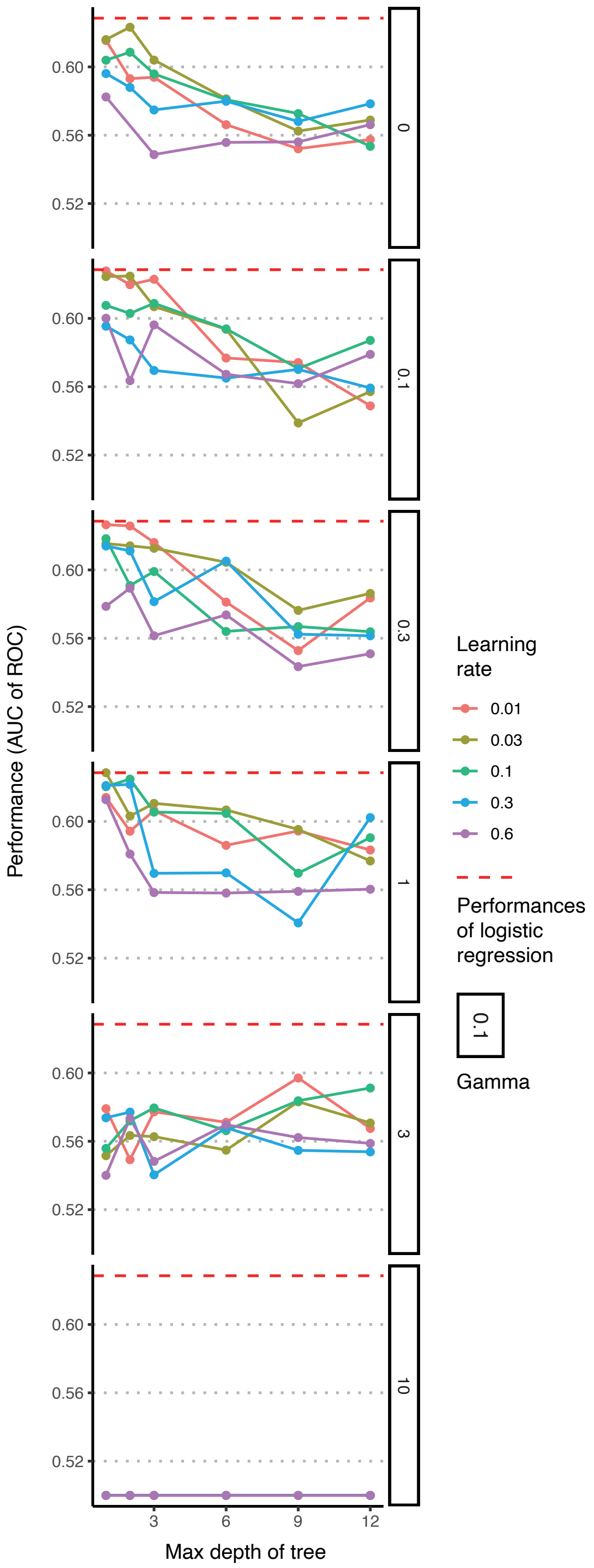

(c)

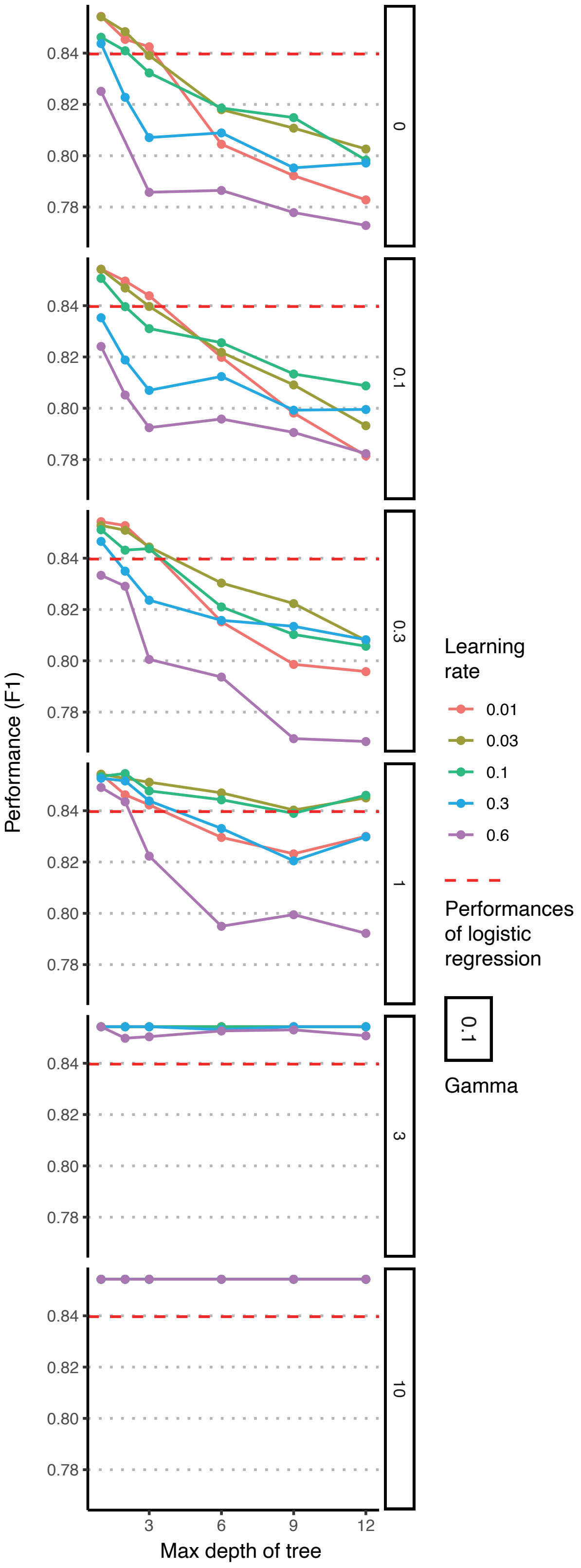

Performance (F1)
Max depth of tree
0.84
0.82
0.80
0.78
3
6
9
12
0
0.1
0.3
1
3
10
Learning rate
0.01
0.03
0.1
0.3
0.6
Performances of logistic regression
0.1
Gamma

(d)

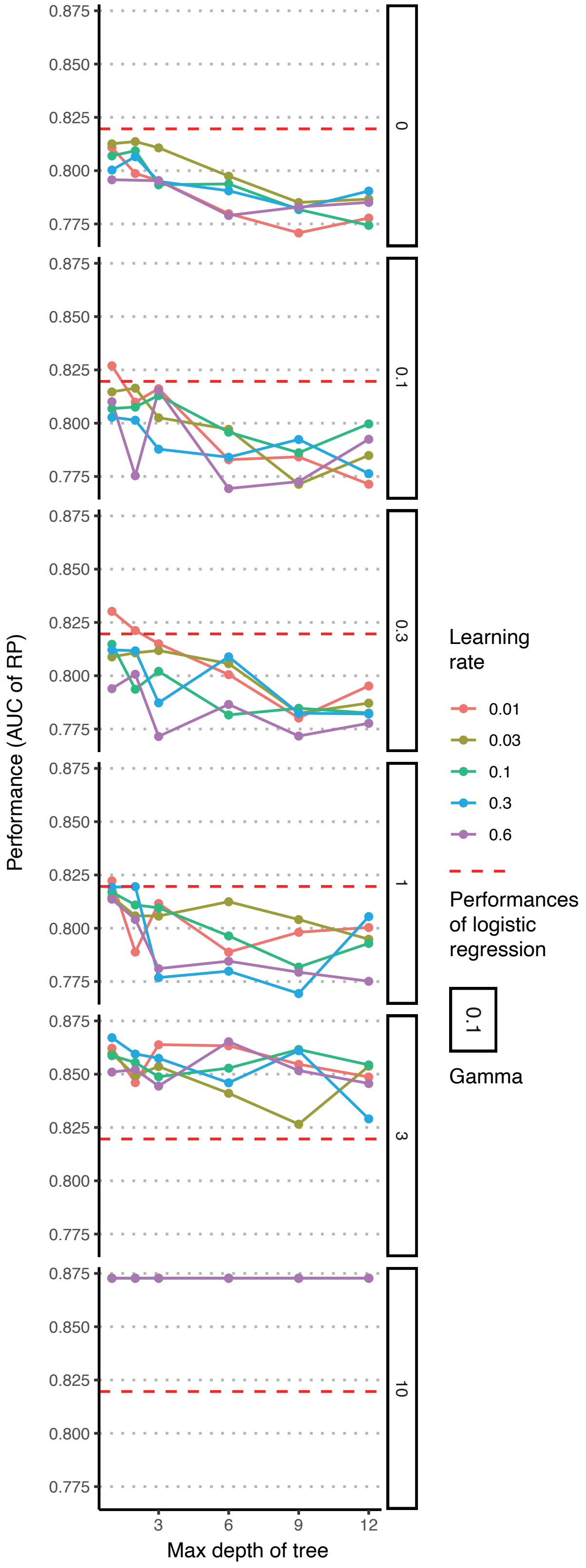

Performance (AUC of RP)
Max depth of tree
0.875
0.850
0.825
0.800
0.775
3
6
9
12
0
0.1
0.3
1
3
10
Learning rate
0.01
0.03
0.1
0.3
0.6
Performances of logistic regression
0.1
Gamma

Figure S3. Prediction performances of ANN with varying numbers of hidden layers, hidden nodes, and learning rates in patients with RA. The X-axis represents the differing numbers of hidden nodes of each hidden layer. The Y-axis represents the (a) accuracy, (b) AUC of the ROC curve, (c) F1 score, and (d) AUC of the precision-recall curve. The performances of the models from each learning rate are depicted as a line. The dashed red horizontal lines represent the prediction performance of logistic regression models.

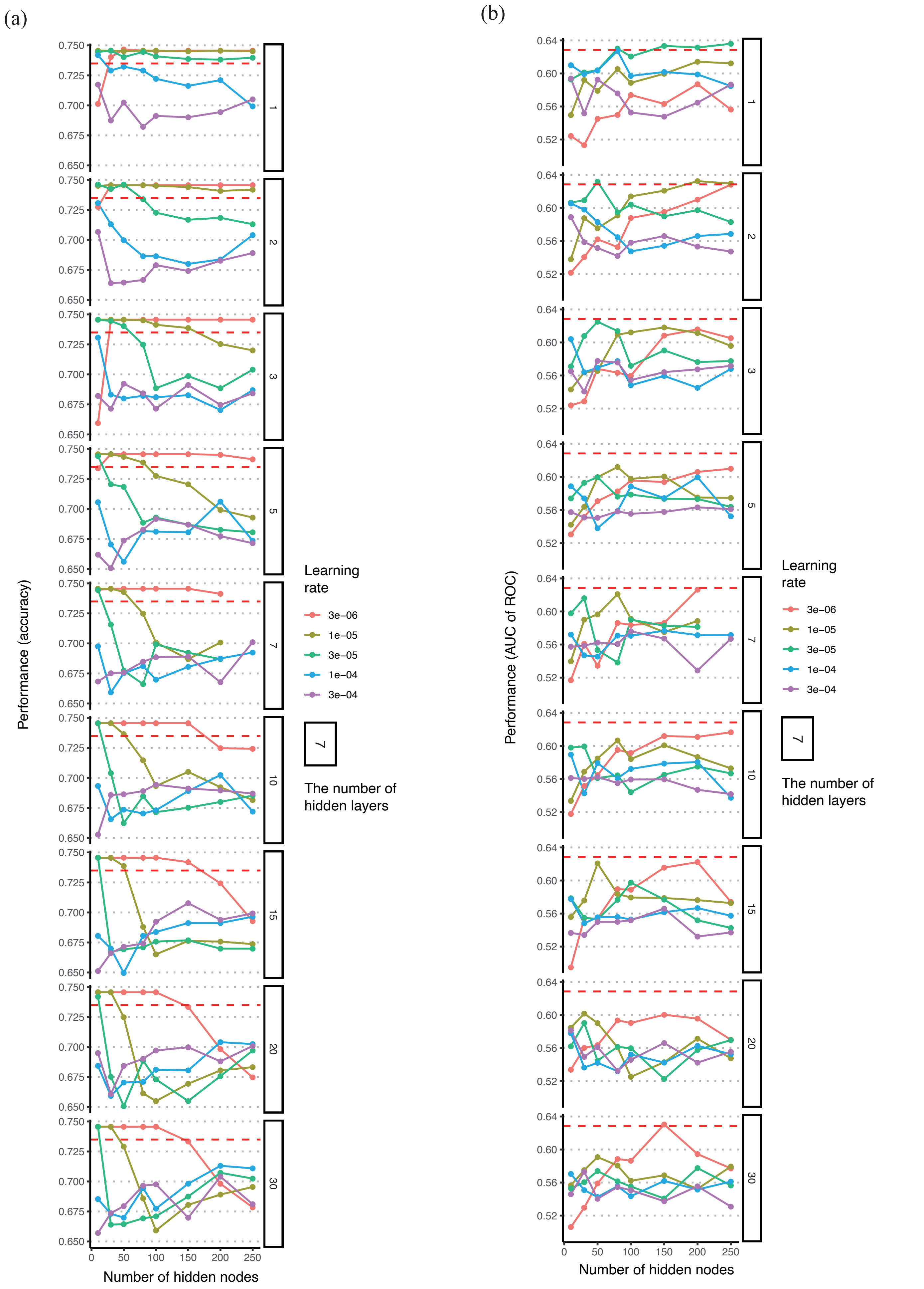

(c)

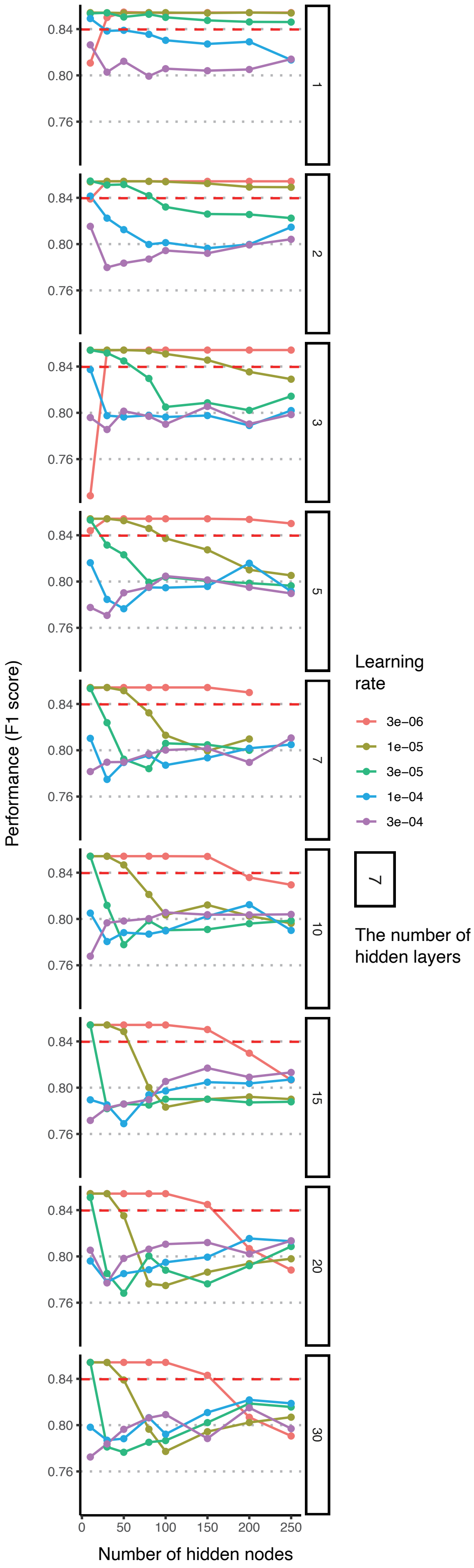
Performance (F1 score)
0.84
0.80
0.76
1
2
3
5
7
10
15
20
30
0
50
100
150
200
250
Number of hidden nodes
Learning rate
3e−06
1e−05
3e−05
1e−04
3e−04
The number of hidden layers

(d)

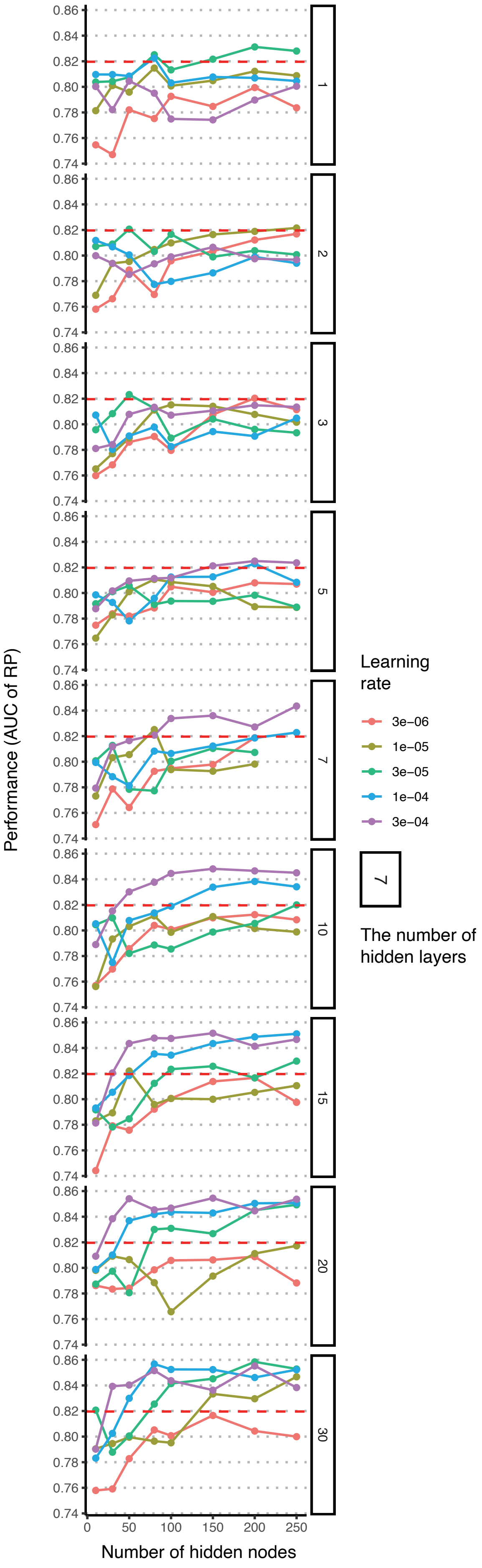
Performance (AUC of RP)
0.86
0.84
0.82
0.80
0.78
0.76
0.74
1
2
3
5
7
10
15
20
30
0
50
100
150
200
250
Number of hidden nodes
Learning rate
3e−06
1e−05
3e−05
1e−04
3e−04
The number of hidden layers

Figure S4. Prediction performances of RF-method model by varying maximum depths of a tree, numbers of trees, minimum sample split, and minimum leaf samples in patients with AS. The X-axis represents the differing maximum depths of a decision tree. The Y-axis represents the (a) accuracy, (b) AUC of the ROC curve, (c) F1 score, and (d) AUC of the precision-recall curve. The performances of the models based on the numbers of trees are depicted as a line. The dashed red horizontal lines represent the prediction performance of logistic regression models.

(a)

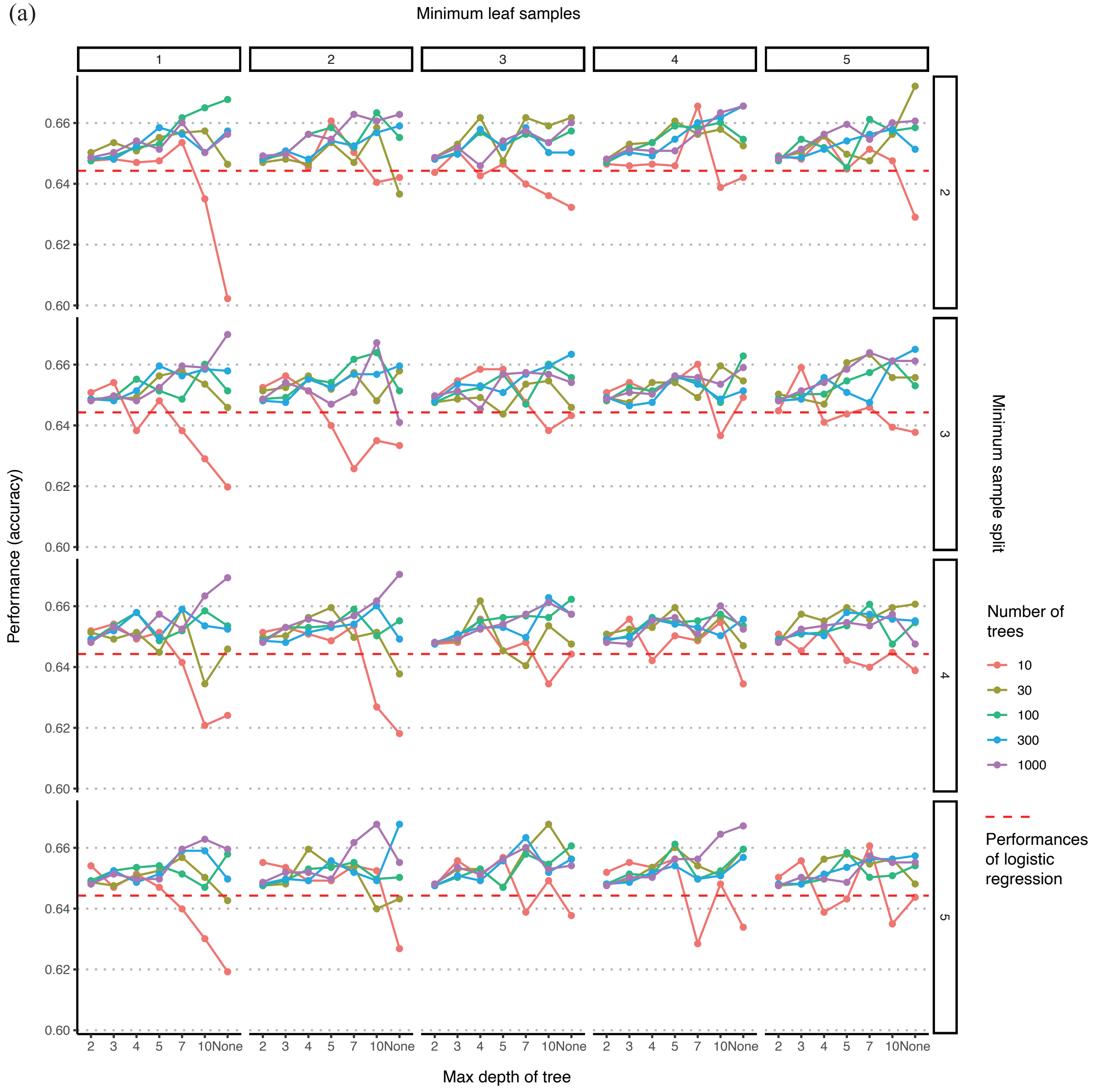

(b)

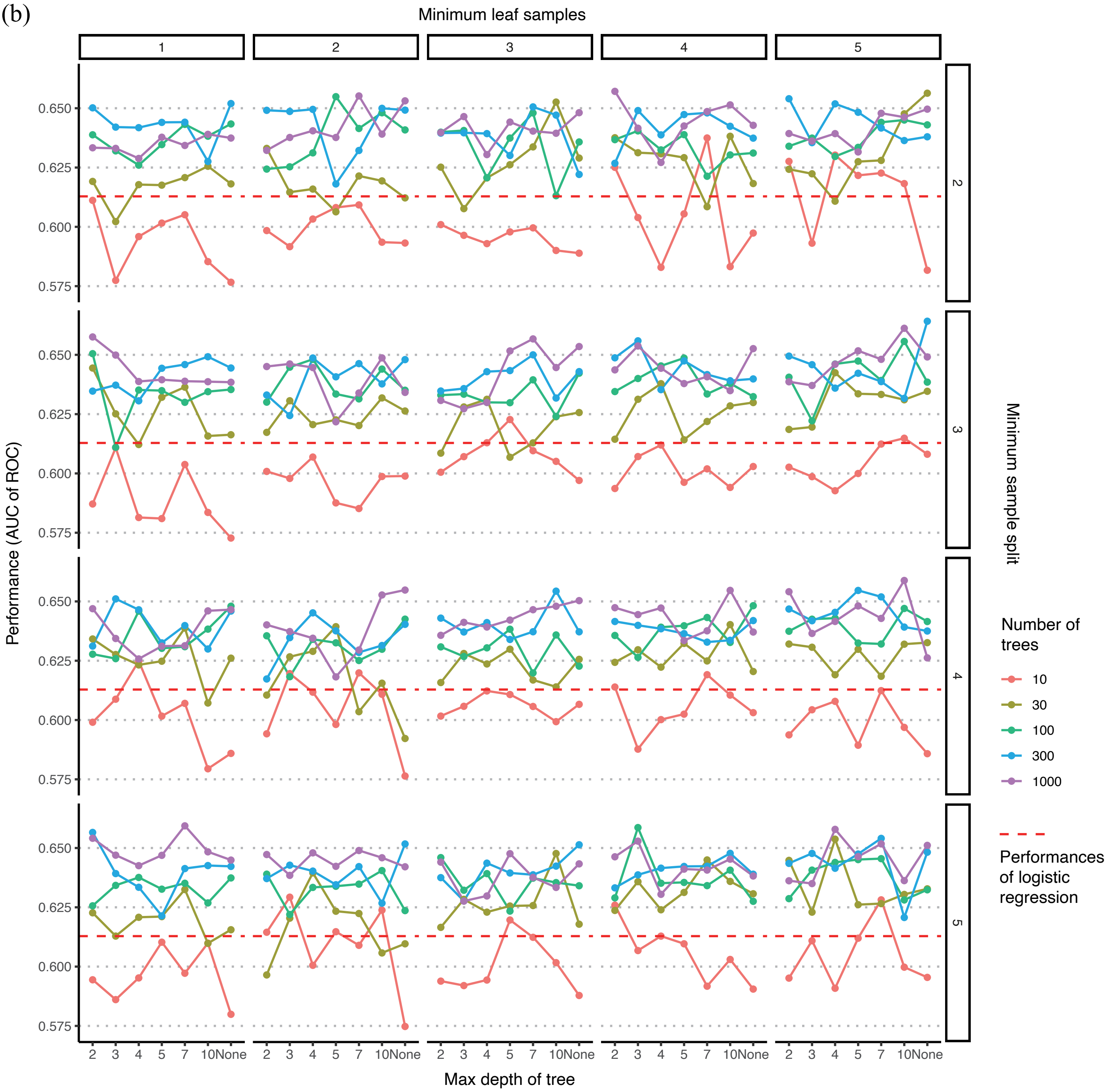

Minimum leaf samples
1
2
3
4
5
Minimum sample split
2
3
4
5
Performance (AUC of ROC)
0.650
0.625
0.600
0.575
Max depth of tree
2 3 4 5 7 10None
Number of trees
10
30
100
300
1000
Performances of logistic regression

(c)

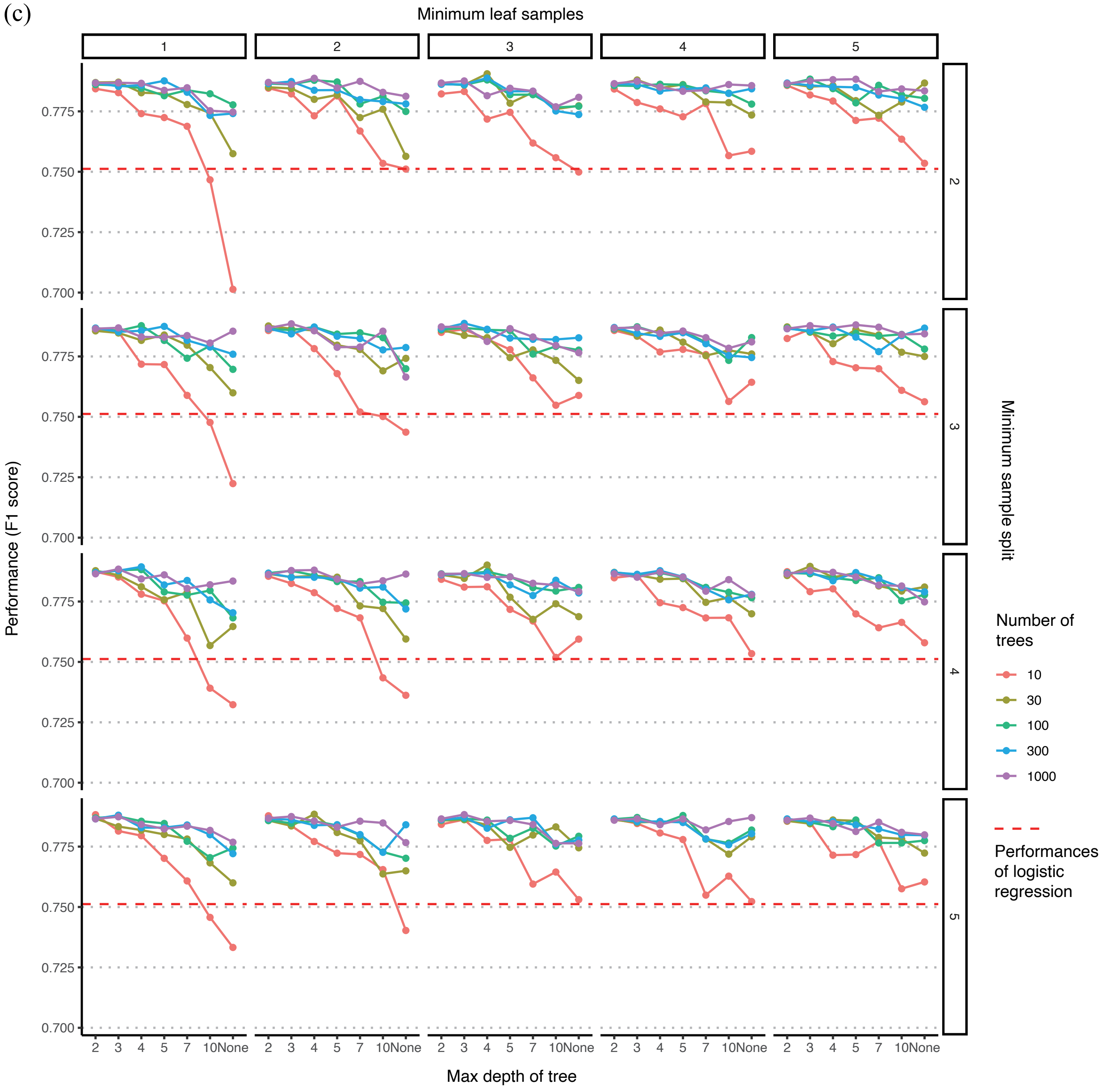

Minimum leaf samples
1
2
3
4
5
Performance (F1 score)
0.700
0.725
0.750
0.775
Minimum sample split
2
3
4
5
Max depth of tree
2 3 4 5 7 10None
Number of trees
10
30
100
300
1000
Performances of logistic regression

(d)

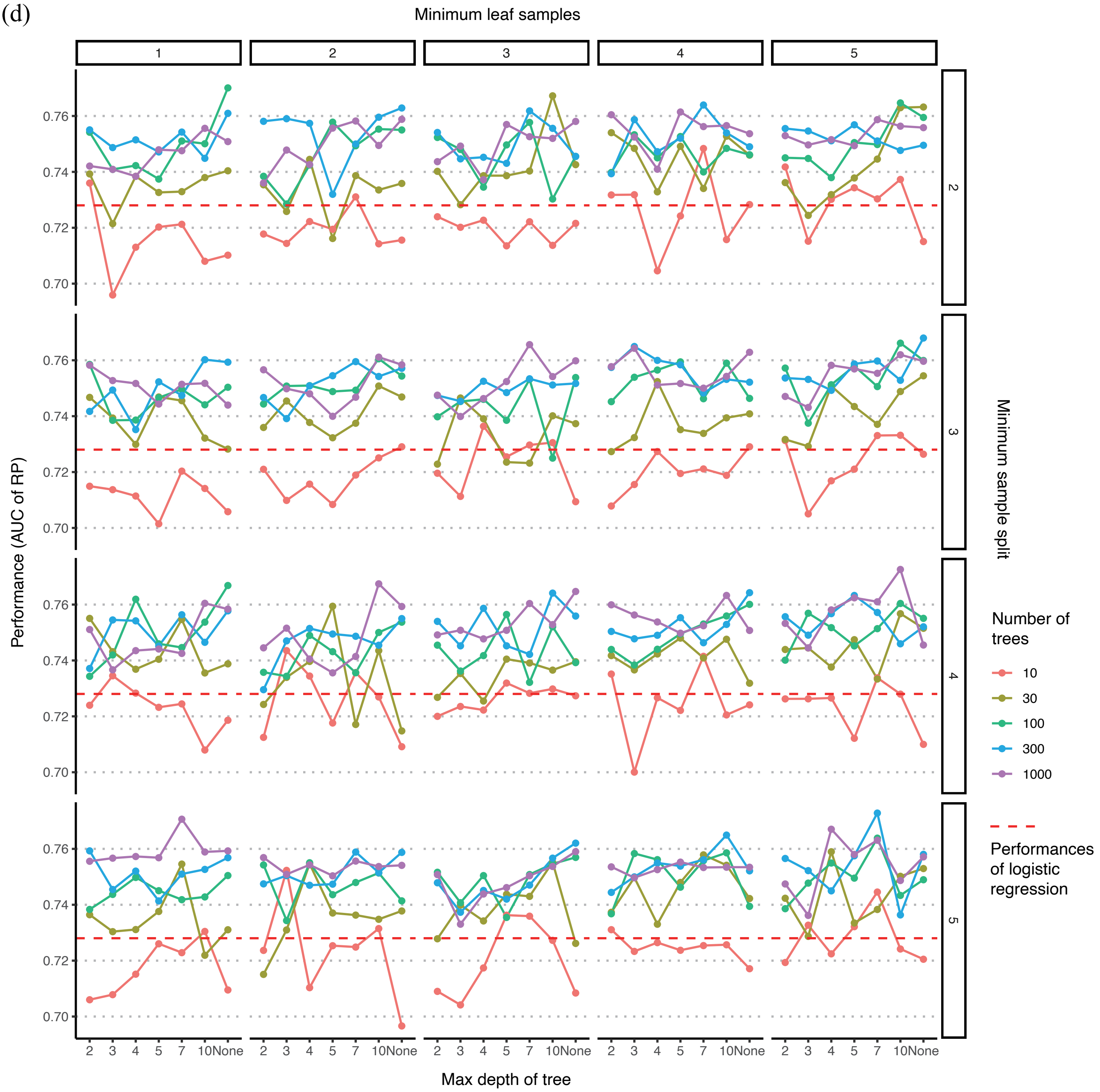

Minimum leaf samples
1
2
3
4
5
Minimum sample split
2
3
4
5
Performance (AUC of RP)
0.70
0.72
0.74
0.76
Max depth of tree
2 3 4 5 7 10None
Number of trees
10
30
100
300
1000
Performances of logistic regression

Figure S5. Prediction performances of XGBoost model by varying maximum depths of a tree and numbers of trees in patients with AS. The X-axis represents the differing maximum depths of a decision tree. The Y-axis represents the (a) accuracy, (b) AUC of the ROC curve, (c) F1 score, and (d) AUC of the precision-recall curve. The performances of the models based on the numbers of trees are depicted as a line. The dashed red horizontal lines represent the prediction performance of logistic regression models.

(a)

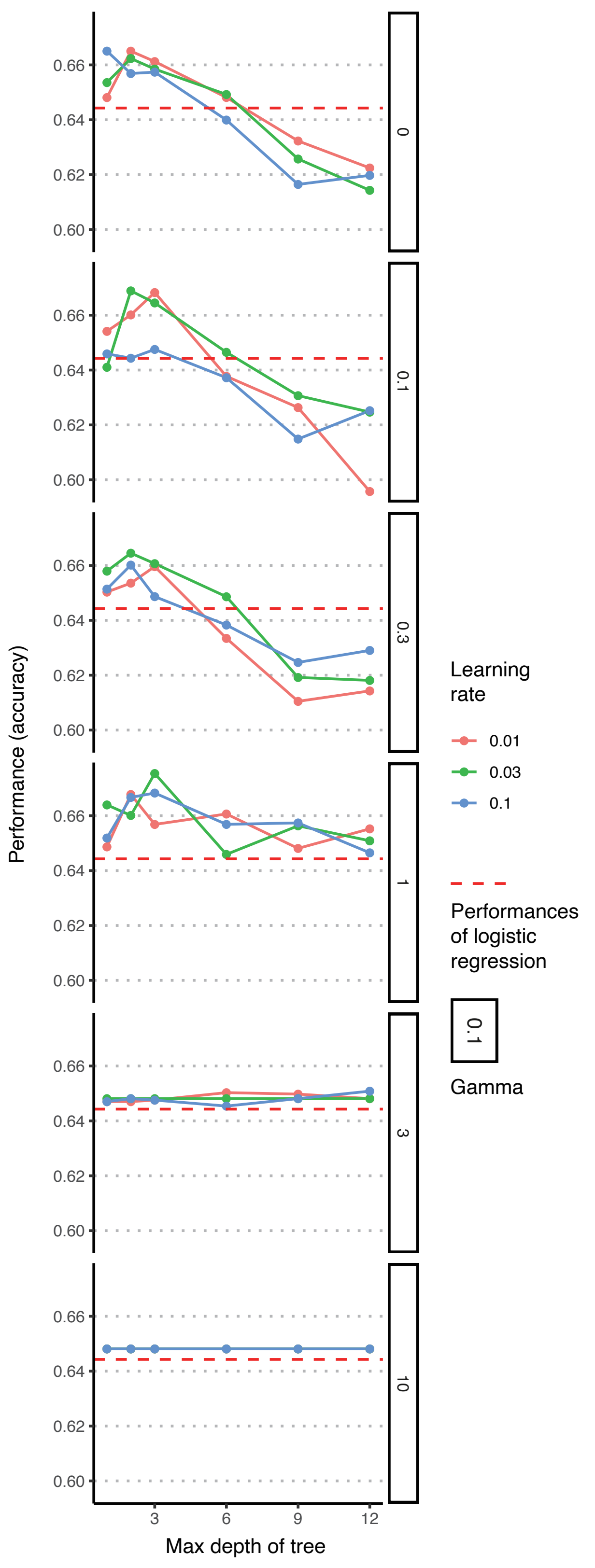

(b)

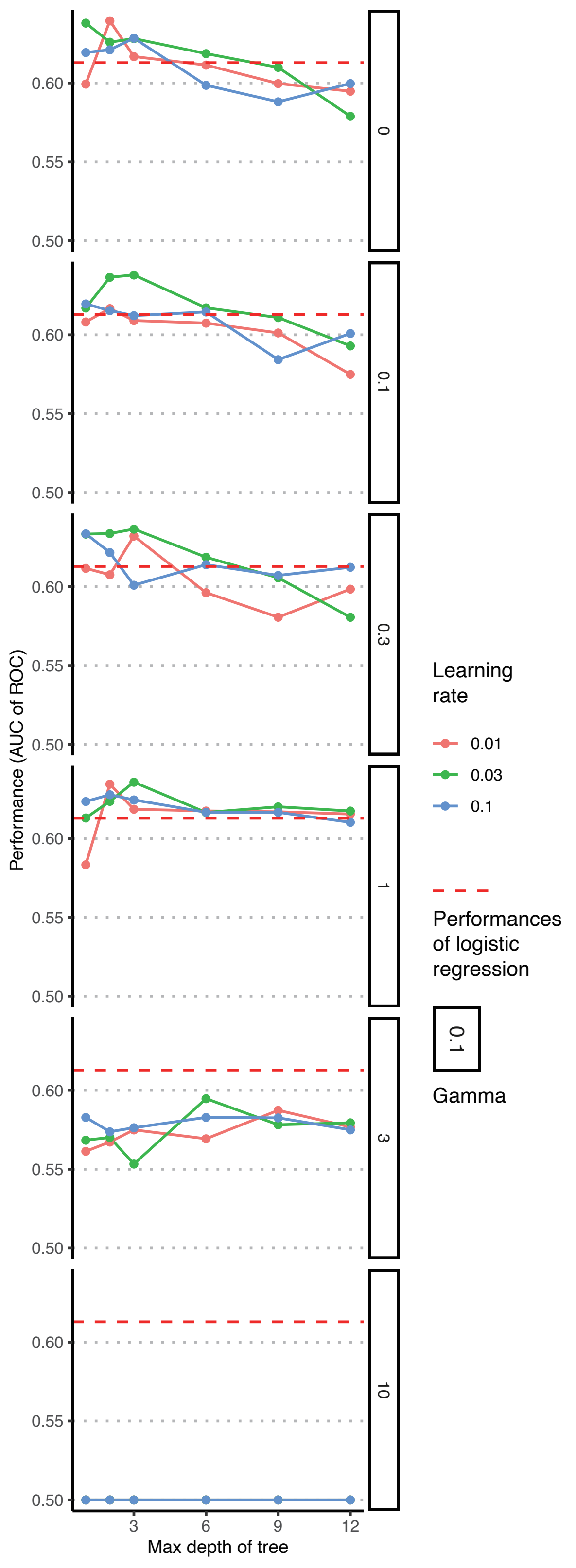

(c)

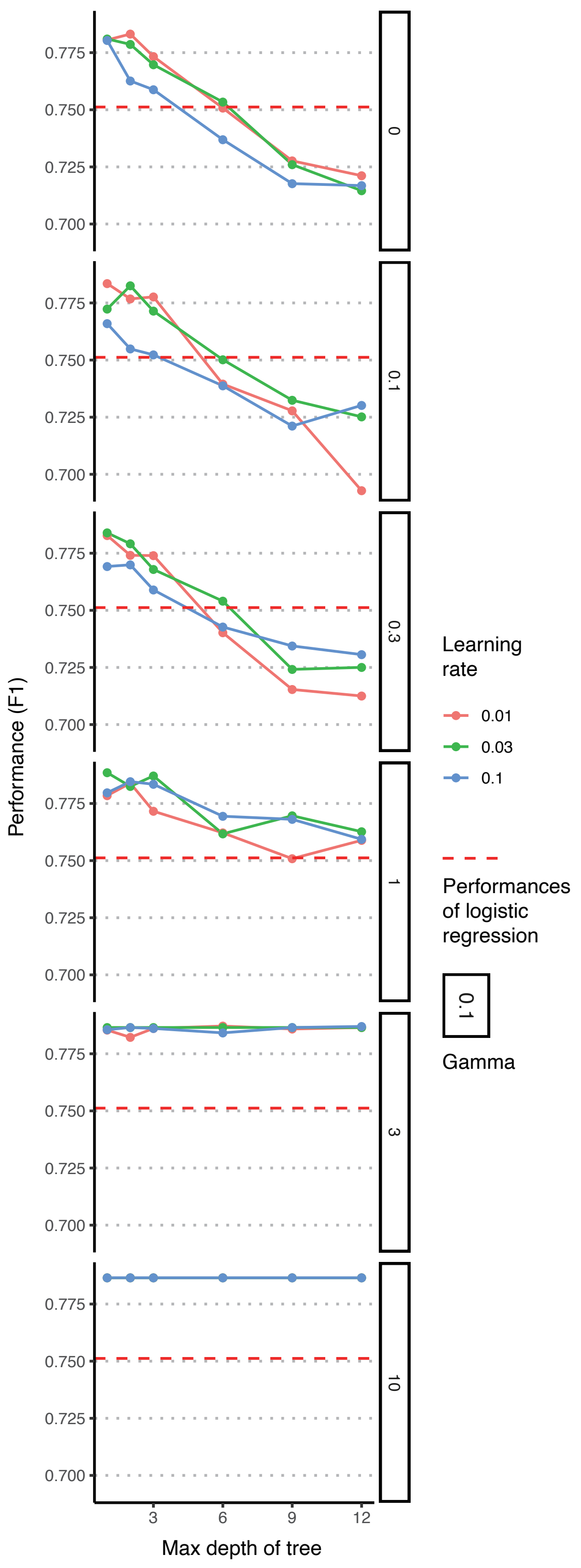
Performance (F1)
0.775
0.750
0.725
0.700
0
0.1
0.3
1
3
10
3
6
9
12
Max depth of tree
Learning rate
0.01
0.03
0.1
Performances of logistic regression
0.1
Gamma

(d)

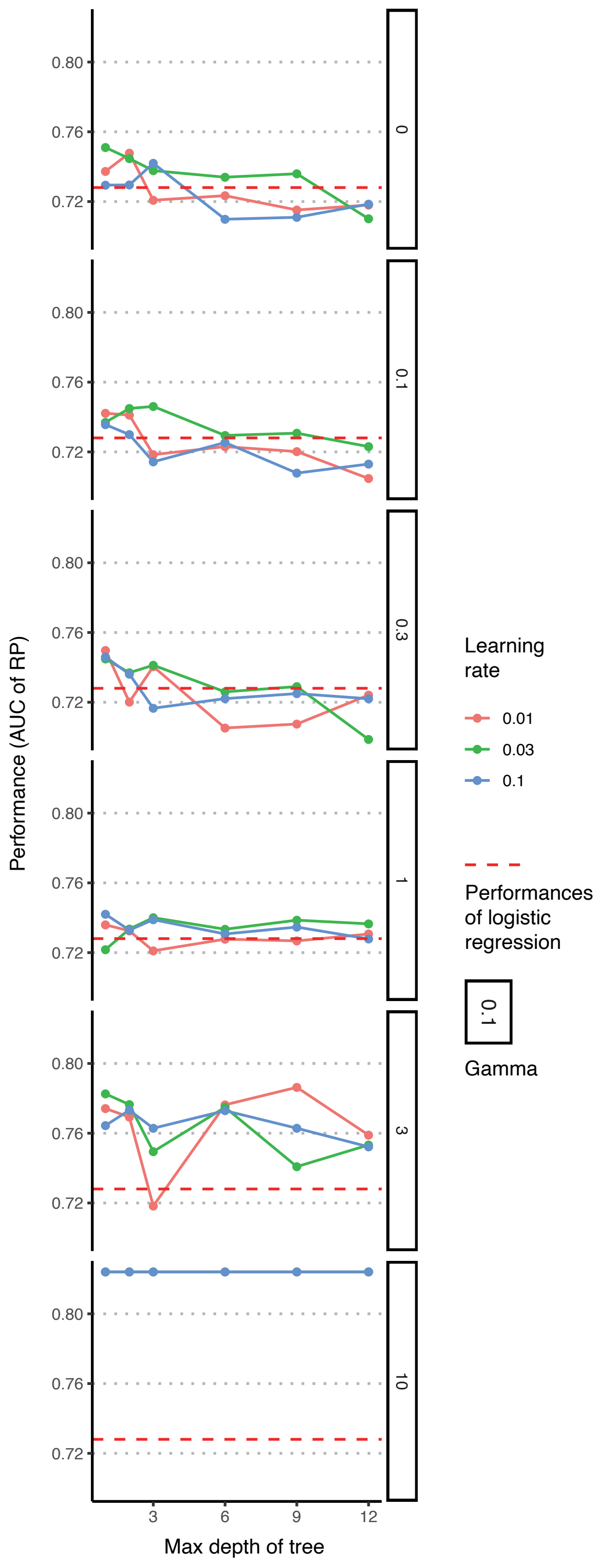
Performance (AUC of RP)
0.80
0.76
0.72
0
0.1
0.3
1
3
10
3
6
9
12
Max depth of tree
Learning rate
0.01
0.03
0.1
Performances of logistic regression
0.1
Gamma

Figure S6. Prediction performances of ANN with varying numbers of hidden layers, hidden nodes, and learning rates in patients with AS. The X-axis represents the differing numbers of hidden nodes of each hidden layer. The Y-axis presents the (a) accuracy, (b) AUC of the ROC curve, (c) F1 score, and (d) AUC of the precision-recall curve. The performances of the models from each learning rate are depicted as a line. The dashed red horizontal lines represent the prediction performance of logistic regression models.

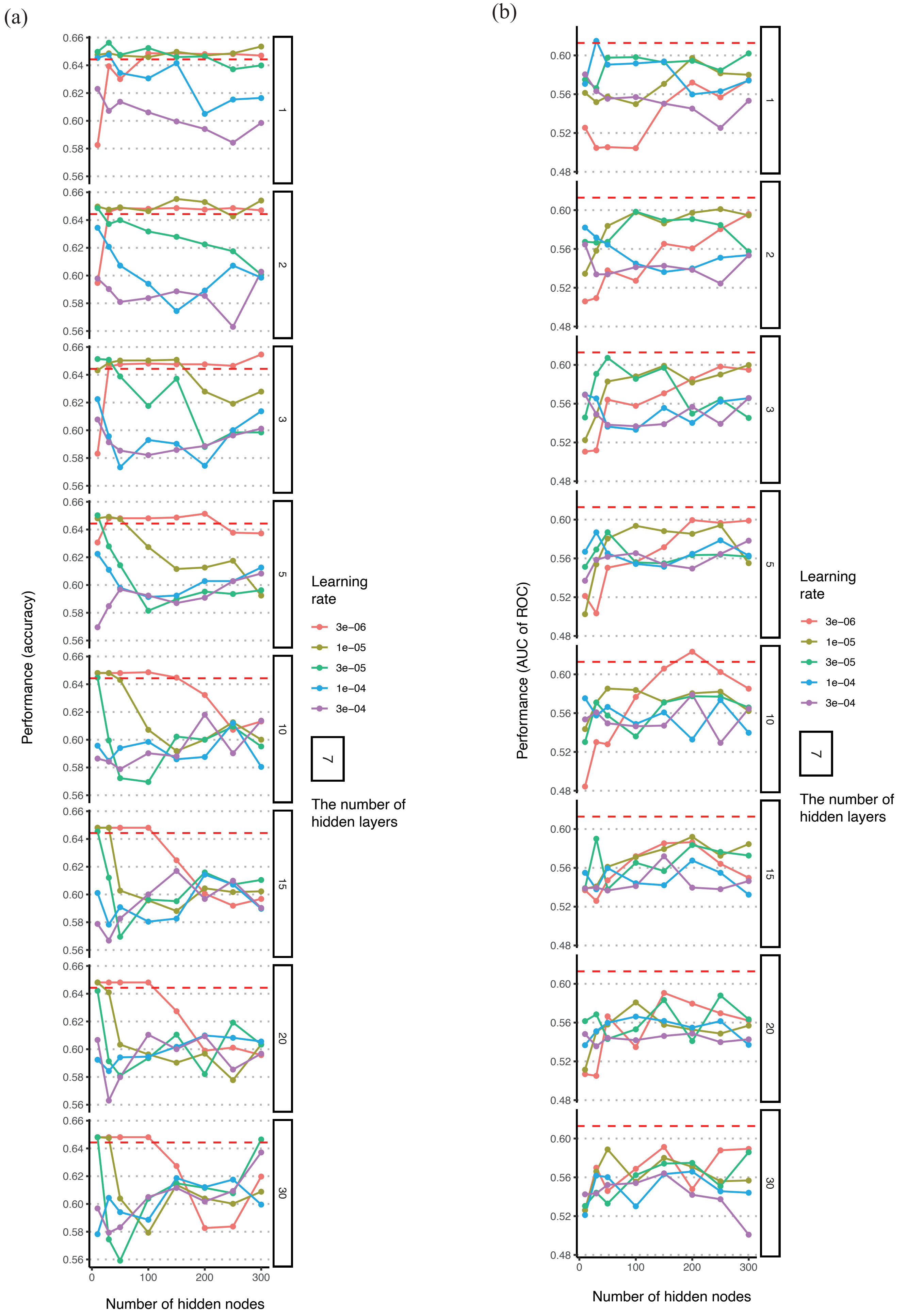

(c)

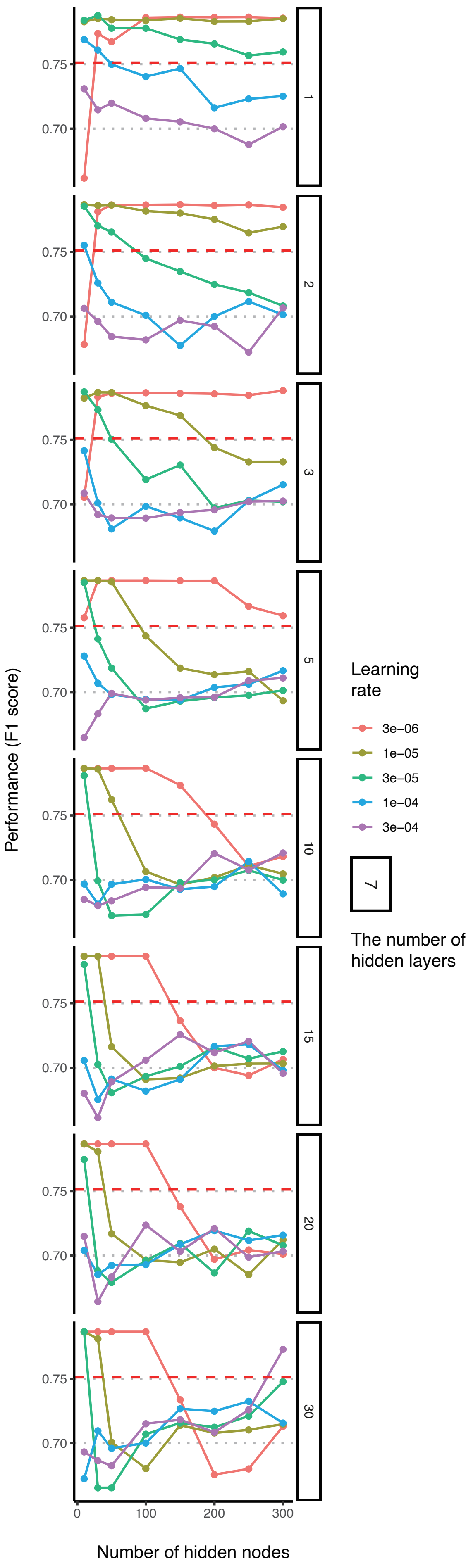

Performance (F1 score)
0.70
0.75
1
2
3
5
10
15
20
30
0
100
200
300
Number of hidden nodes
Learning rate
3e−06
1e−05
3e−05
1e−04
3e−04
7
The number of hidden layers

(d)

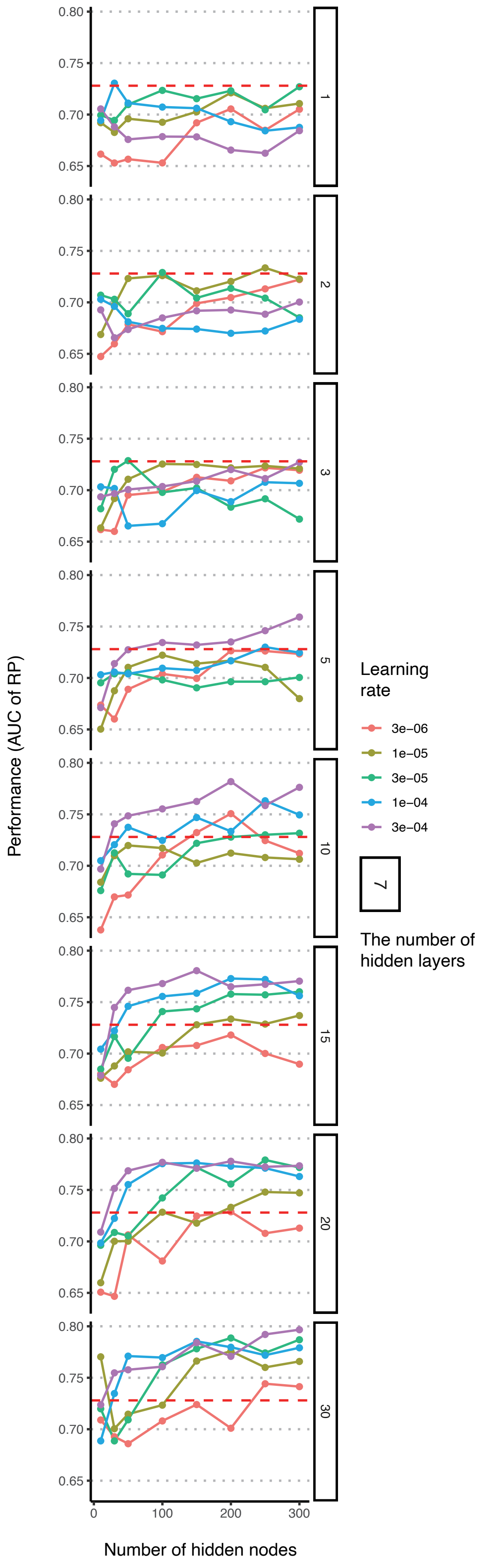

Performance (AUC of RP)
0.65
0.70
0.75
0.80
1
2
3
5
10
15
20
30
0
100
200
300
Number of hidden nodes
Learning rate
3e−06
1e−05
3e−05
1e−04
3e−04
7
The number of hidden layers

Figure S7. Performance of RF-method and logistic regression models in RA patients divided by the type of bDMARDs, (a) by training dataset, and (b) by independent test dataset.

(a)

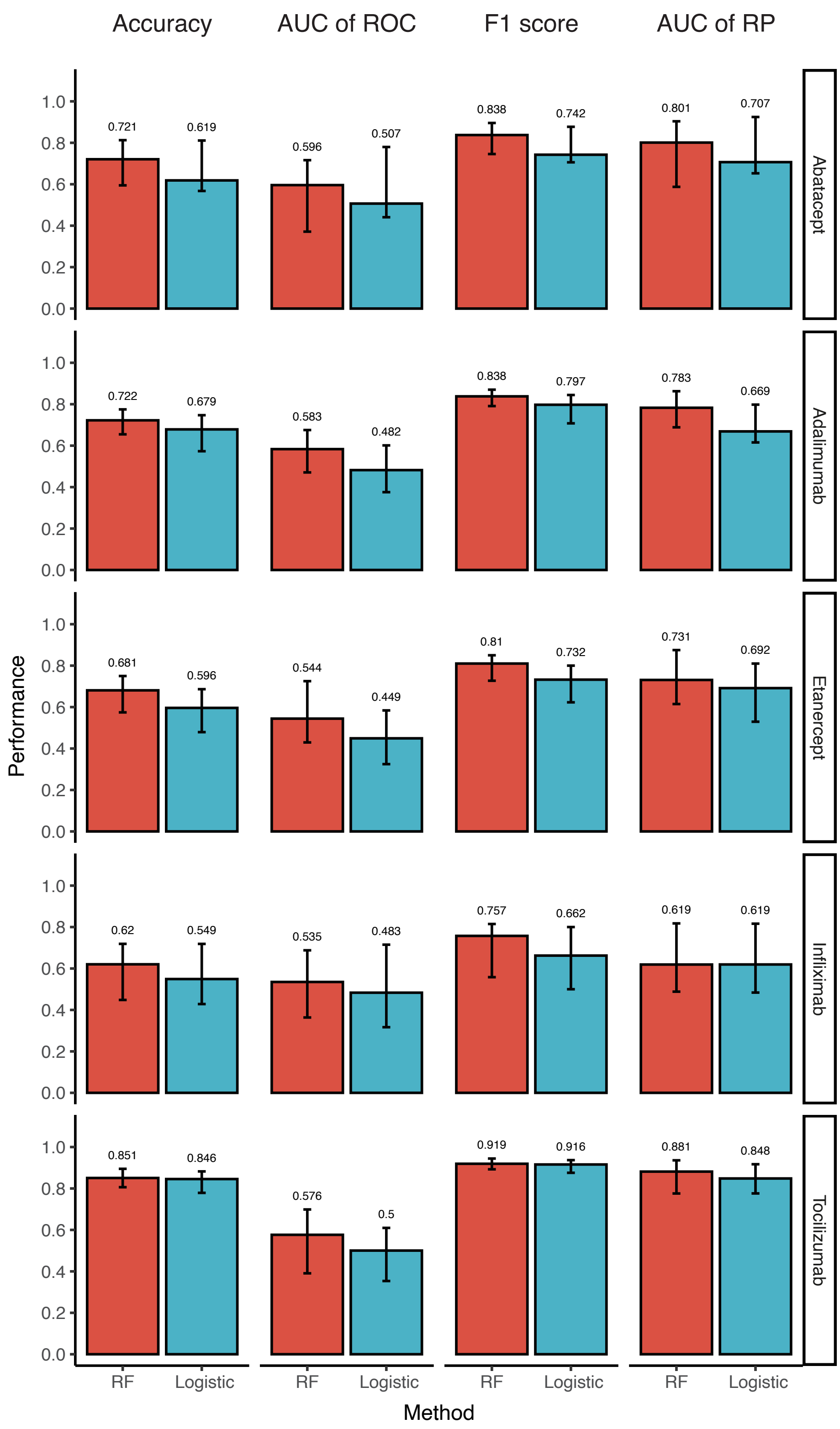

(b)

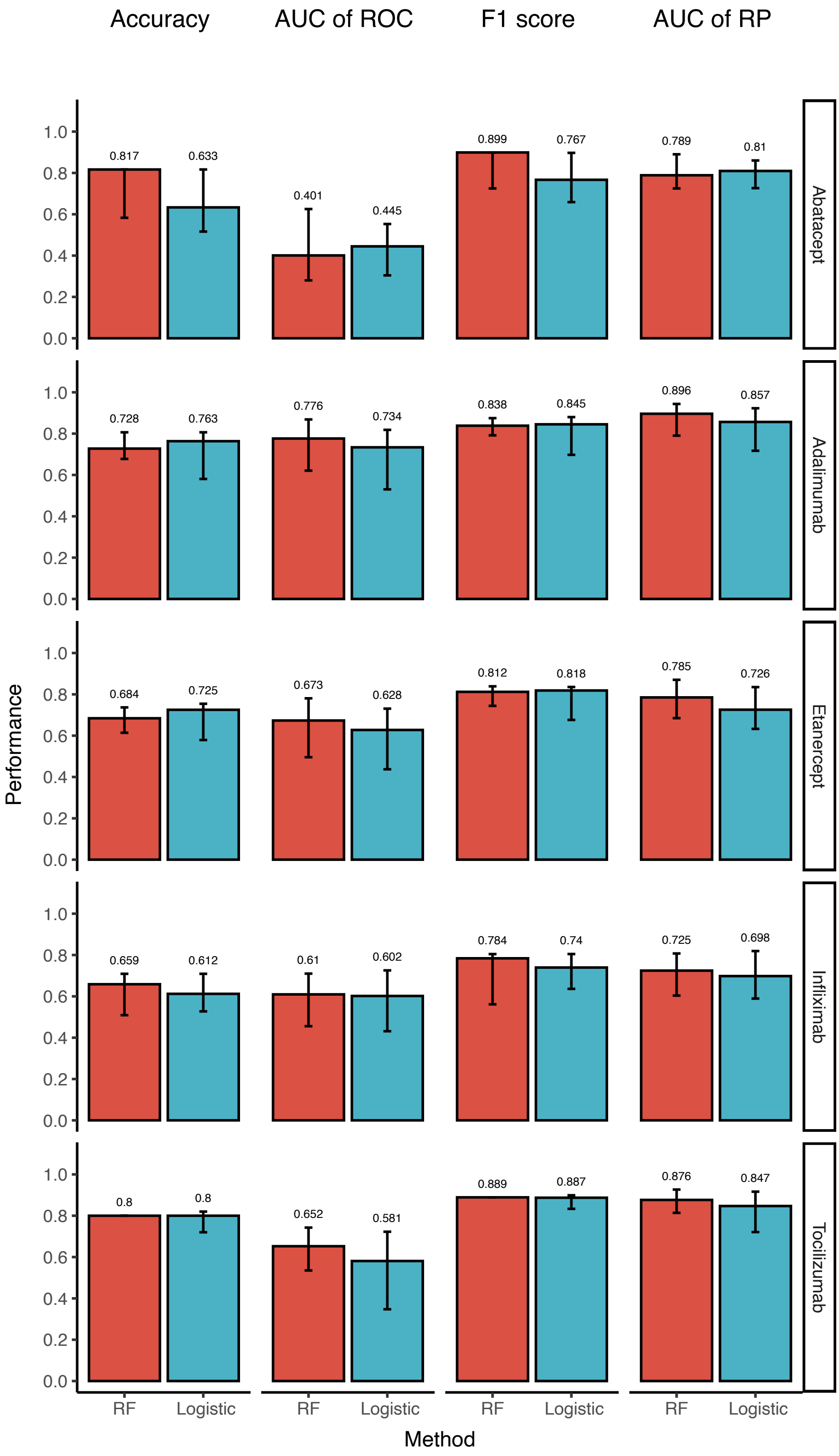

Accuracy
AUC of ROC
F1 score
AUC of RP
Abatacept
Adalimumab
Etanercept
Infliximab
Tocilizumab
Performance
Method
RF
Logistic
0.0
0.2
0.4
0.6
0.8
1.0
0.817
0.633
0.401
0.445
0.899
0.767
0.789
0.81
0.728
0.763
0.776
0.734
0.838
0.845
0.896
0.857
0.684
0.725
0.673
0.628
0.812
0.818
0.785
0.726
0.659
0.612
0.61
0.602
0.784
0.74
0.725
0.698
0.8
0.8
0.652
0.581
0.889
0.887
0.876
0.847

Figure S8. Result of the feature importance analysis of the best-performing RF-method model in RA patients divided by the type of bDMARDs. (a) Abatacept, (b) adalimumab, (c) etanercept, (d) infliximab, and (e) tocilizumab.

(a) abatacept

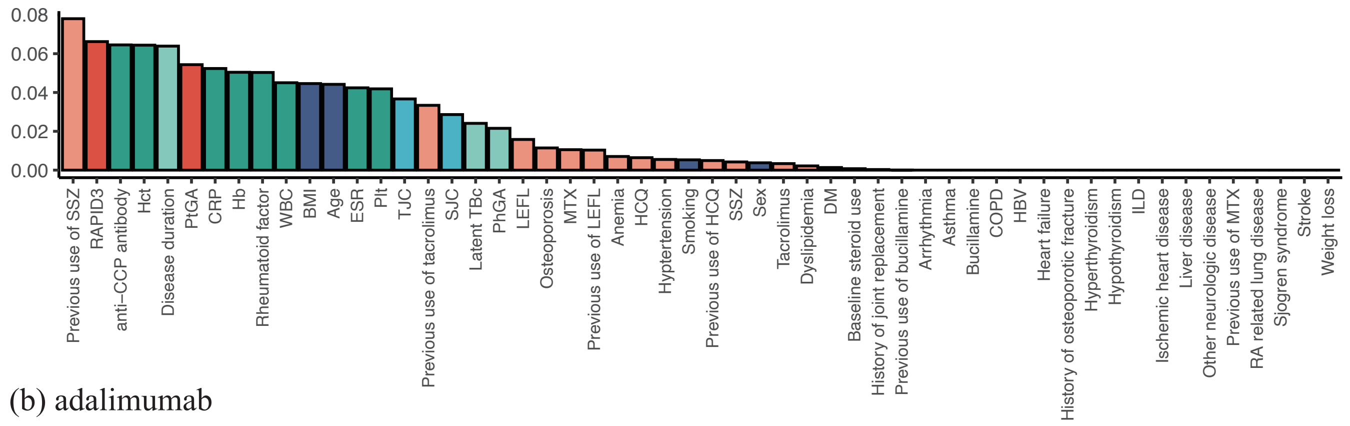

(b) adalimumab

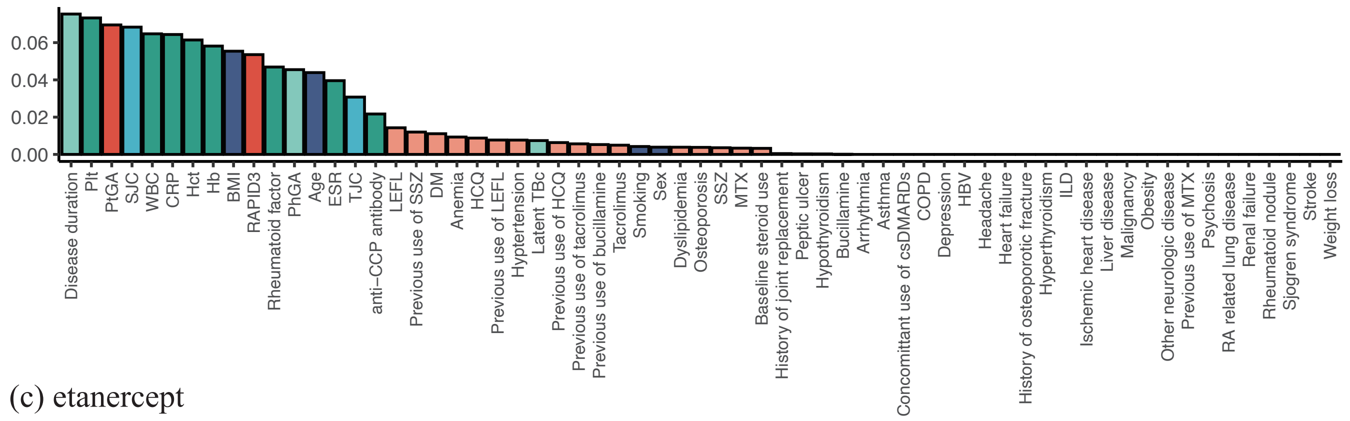

(c) etanercept

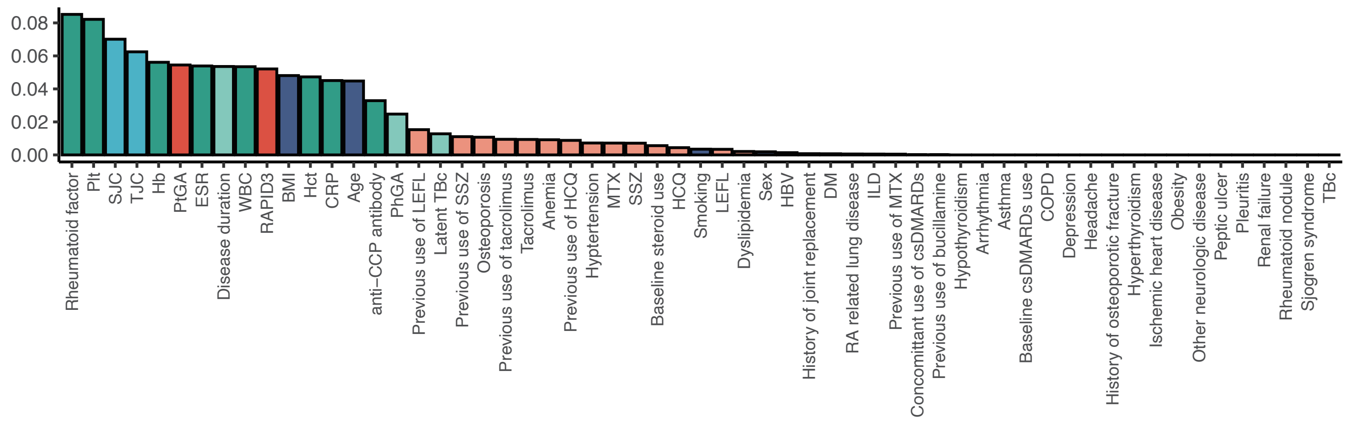

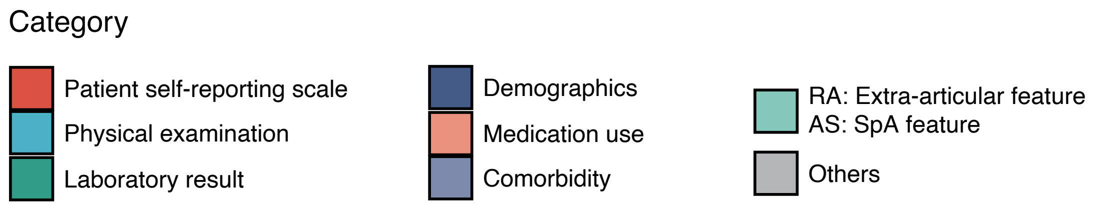

(d) etanercept

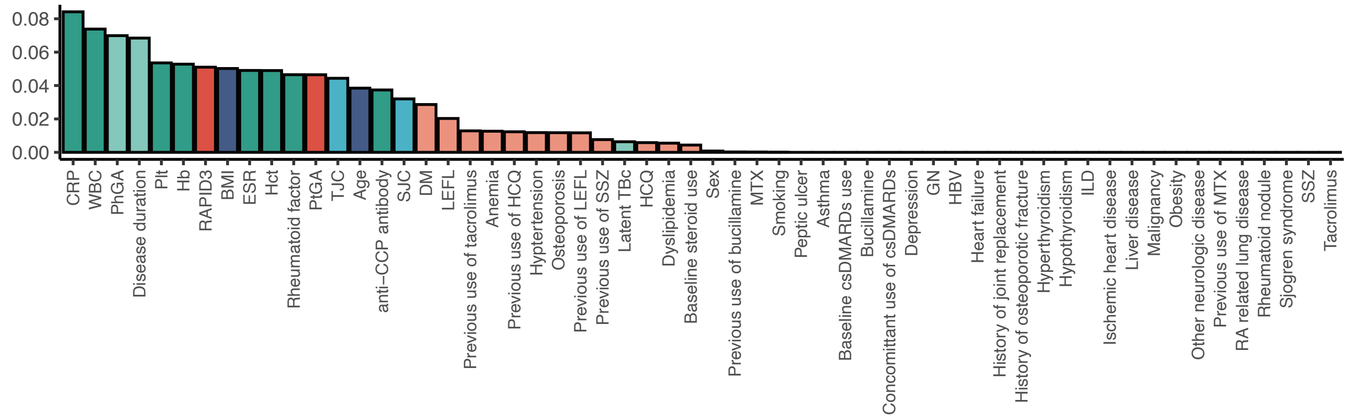

(e) tocilizumab

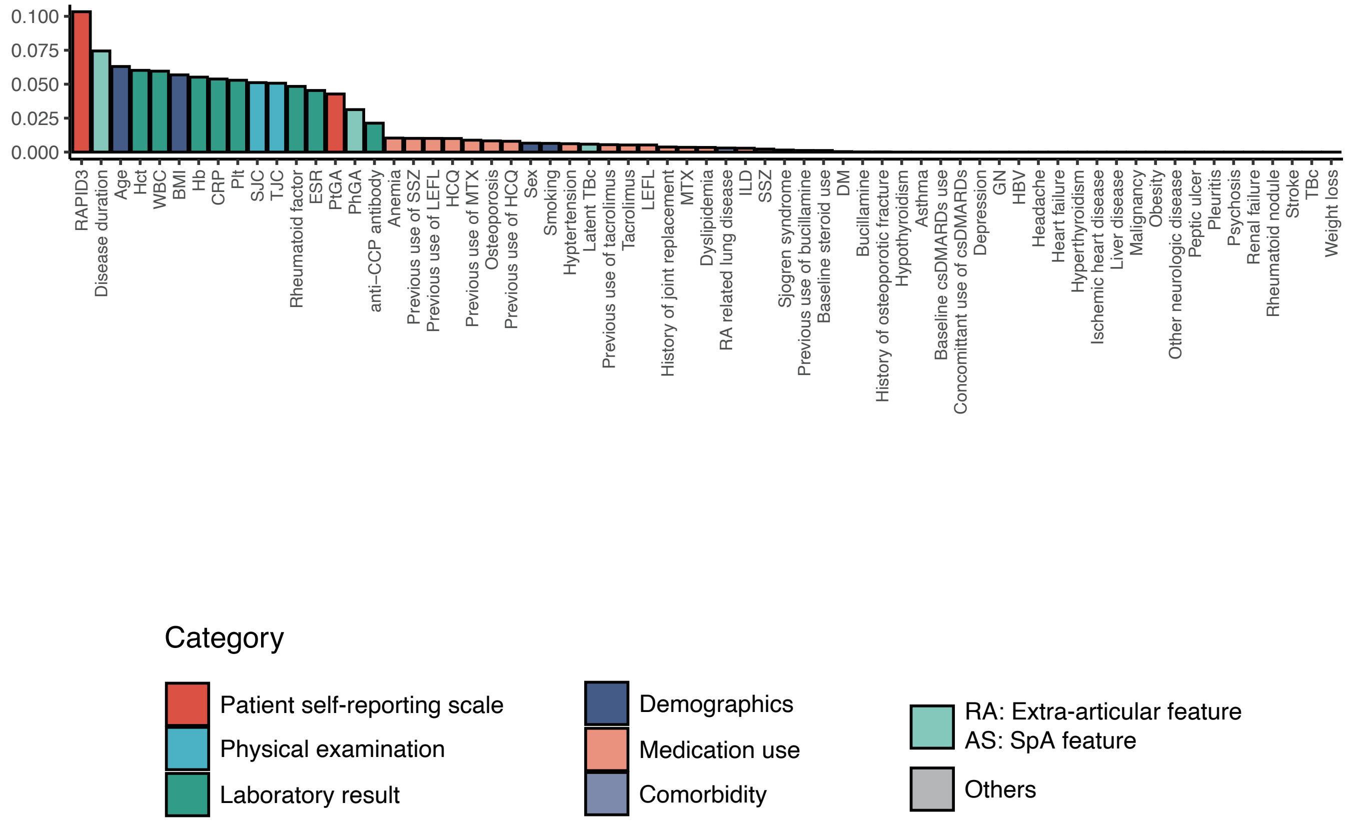

Figure S9. Performance of RF and logistic regression models in AS patients divided by the type of bDMARDs, (a) by training dataset, and (b) by independent test dataset.

(a)

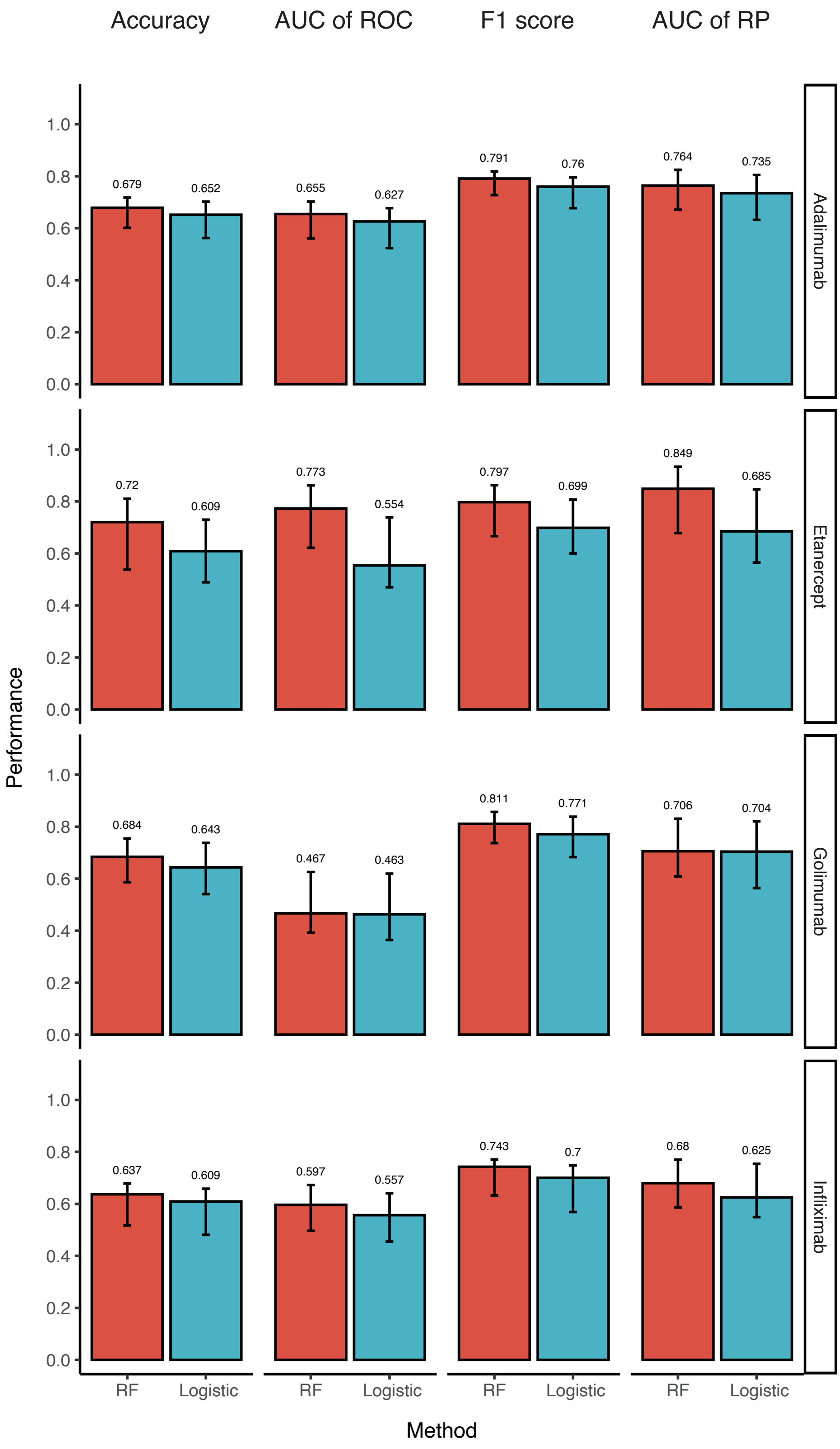

(b)

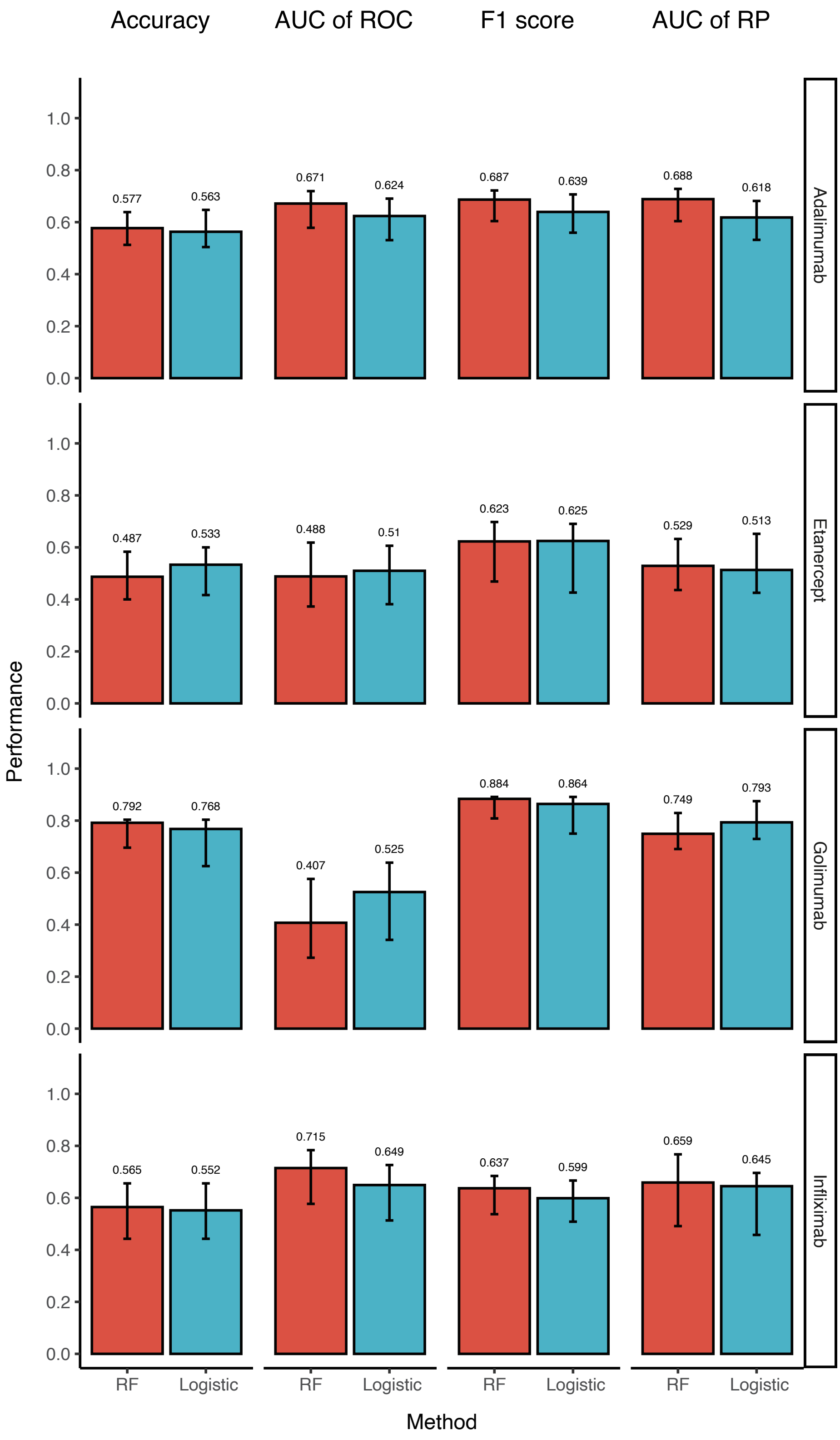
Accuracy
AUC of ROC
F1 score
AUC of RP
Adalimumab
Etanercept
Golimumab
Infliximab
Performance
Method
RF
Logistic
0.577
0.563
0.671
0.624
0.687
0.639
0.688
0.618
0.487
0.533
0.488
0.51
0.623
0.625
0.529
0.513
0.792
0.768
0.407
0.525
0.884
0.864
0.749
0.793
0.565
0.552
0.715
0.649
0.637
0.599
0.659
0.645

Figure S10. Feature importance analysis of the best-performing RF-method model in AS patients divided by the type of bDMARDs. (a) Adalimumab, (b) etanercept, (c) golimumab, and (d) infliximab.

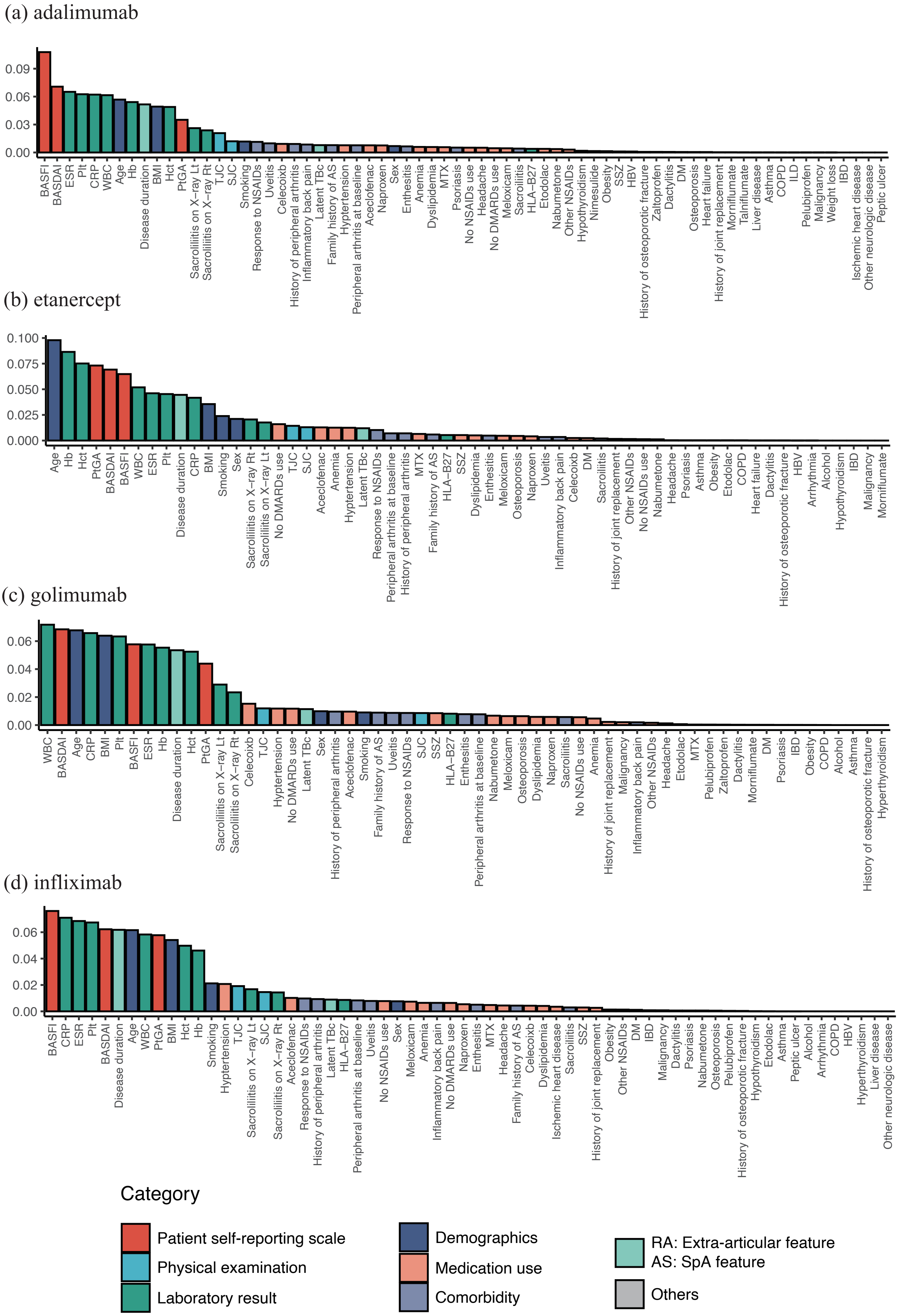

Figure S11. Correlation analysis between input features that reported the feature importance analysis. (a) RA, (b) AS.

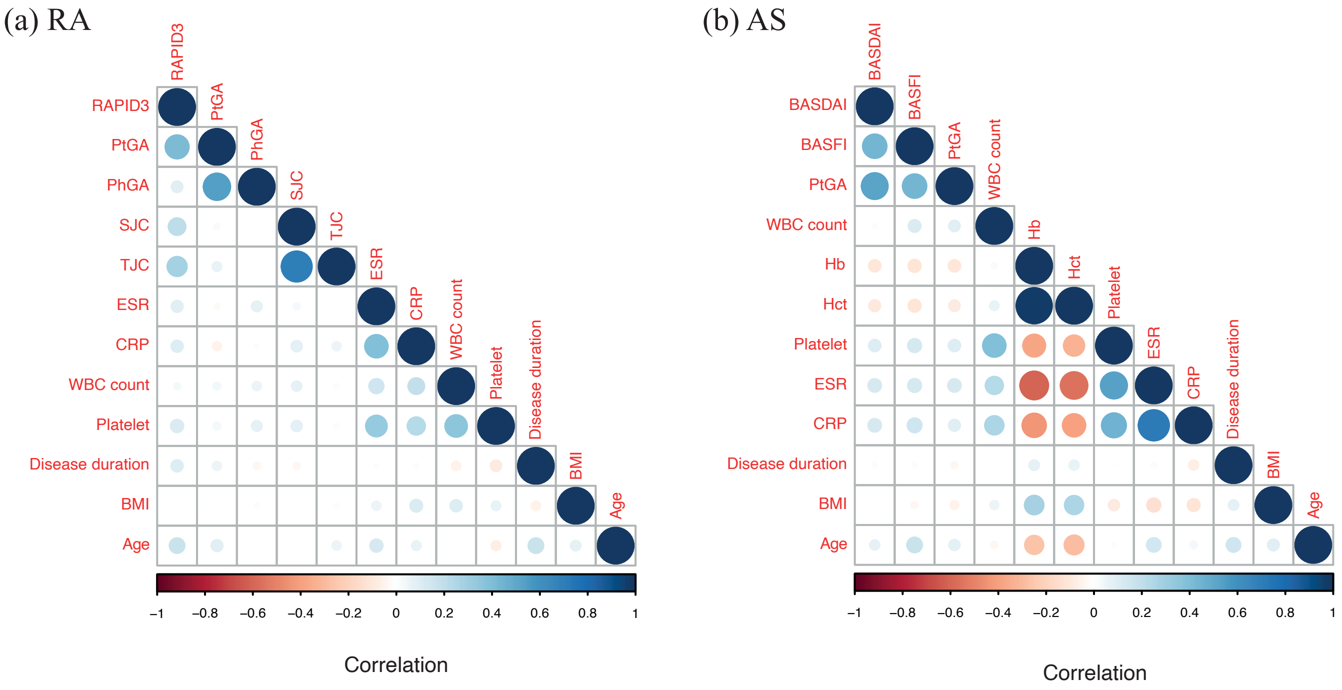

Figure S12. Linear regression analysis with dot plot results for (a) RAPID3 and other features, (b) PtGA and other features in patients with RA; between (c) BASFI and other features, (d) BASDAI and other features, (e) PtGA and other features in patients with AS; (f) between patient self-reporting scales in both diseases.

(a) RAPID3 and other features in patients with RA

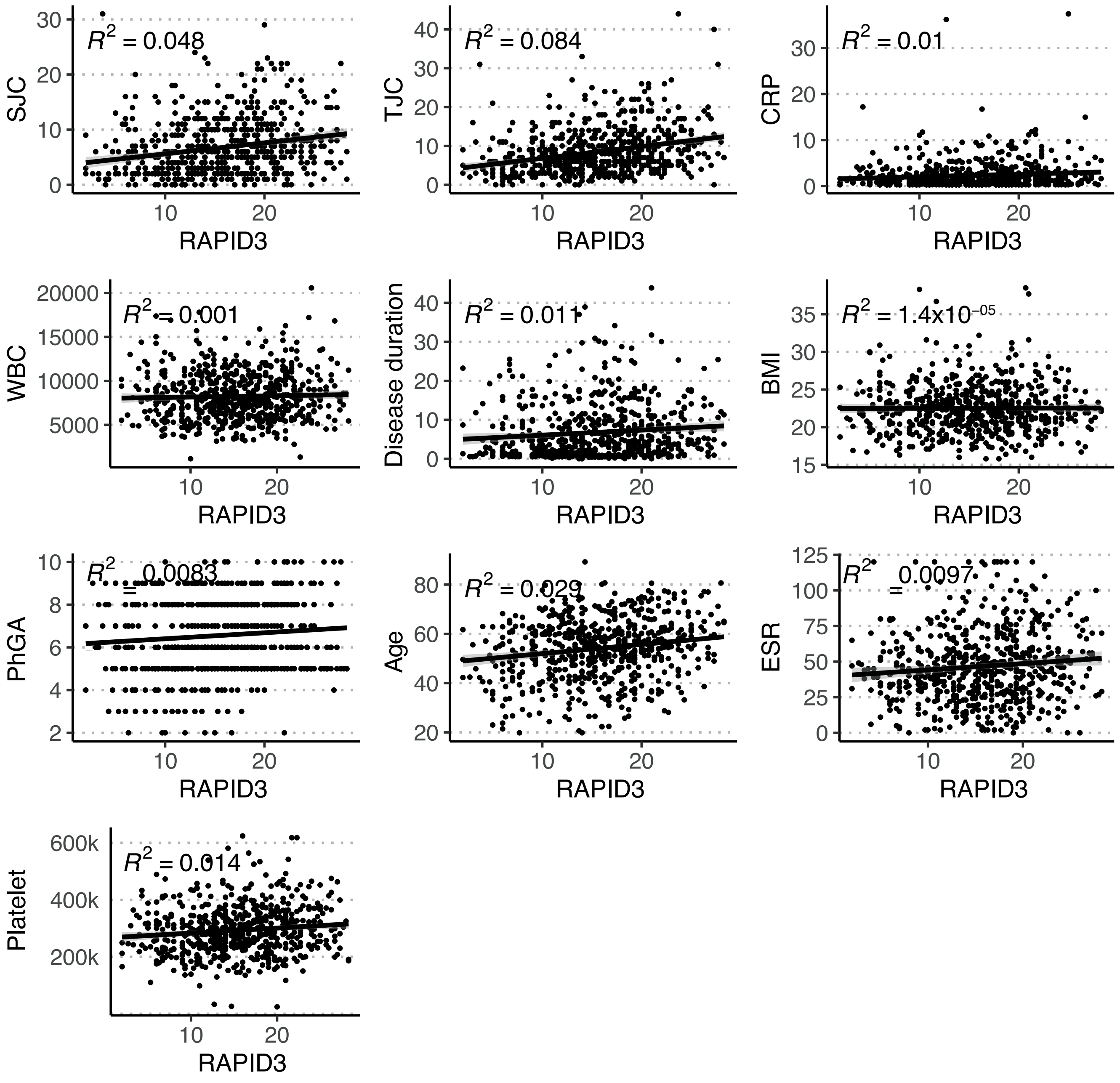

(b) PtGA and other features in patients with RA

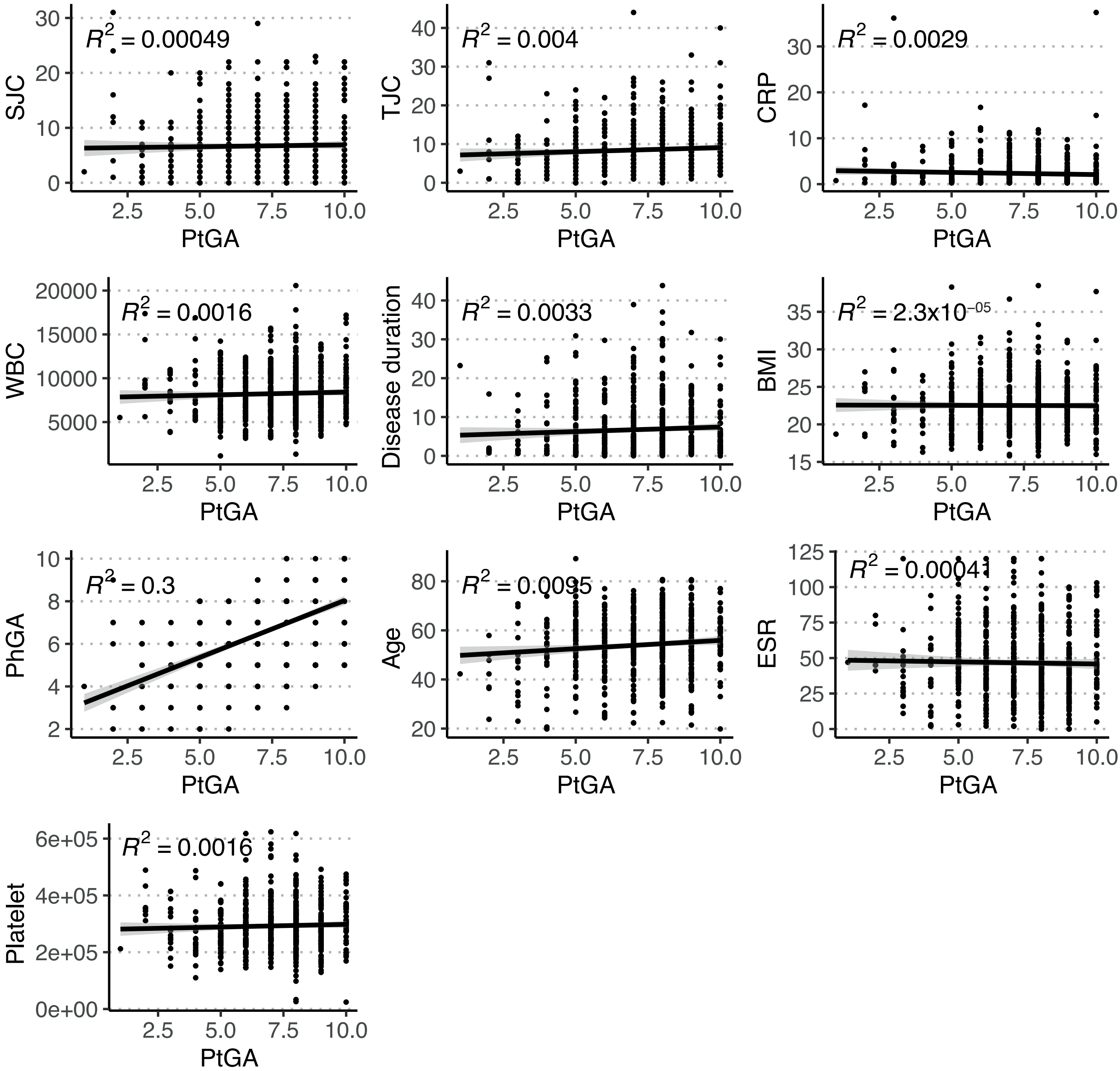

(c) BASFI and other features in patients with AS

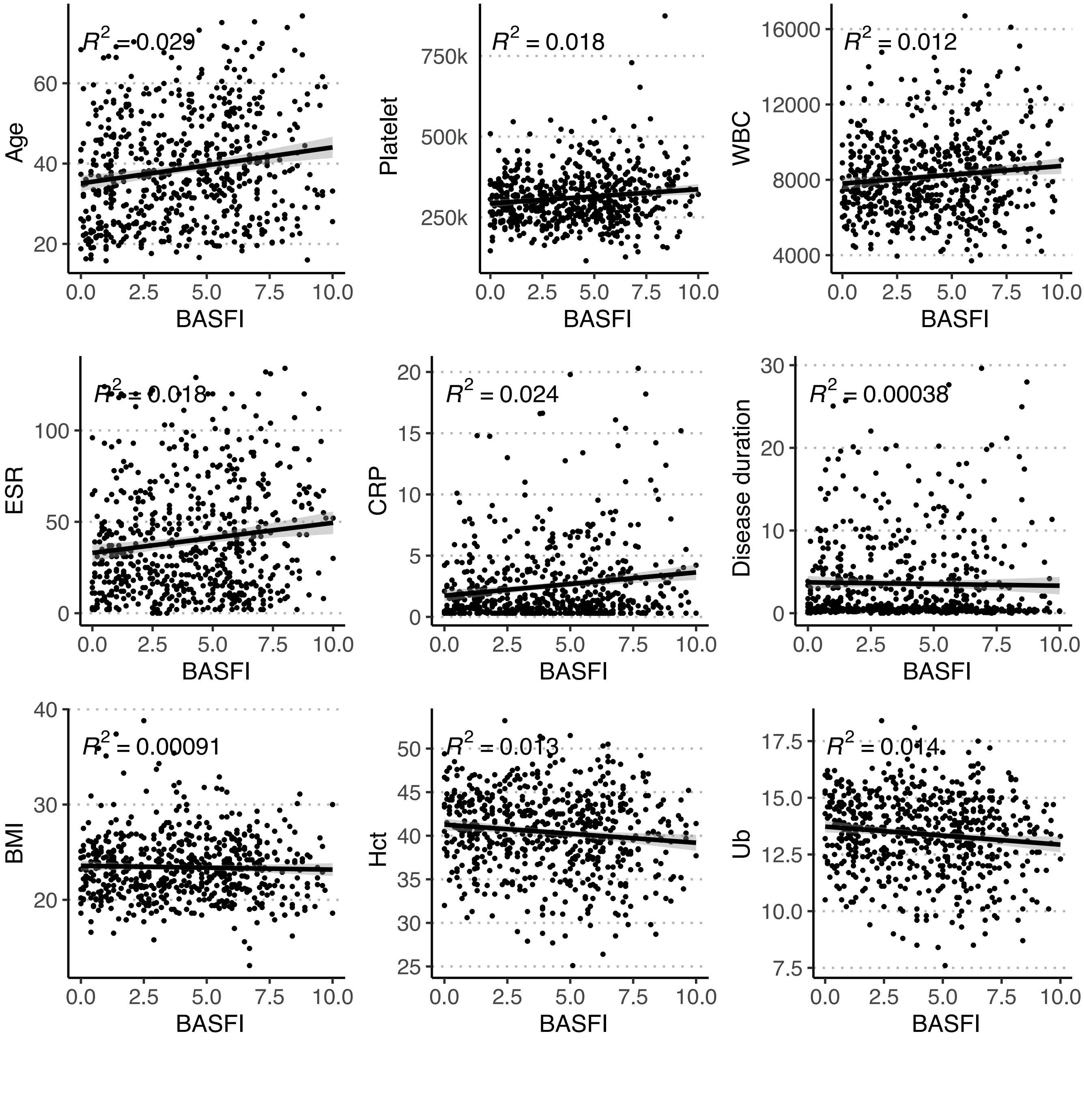

(d) BASDAI and other features in patients with AS

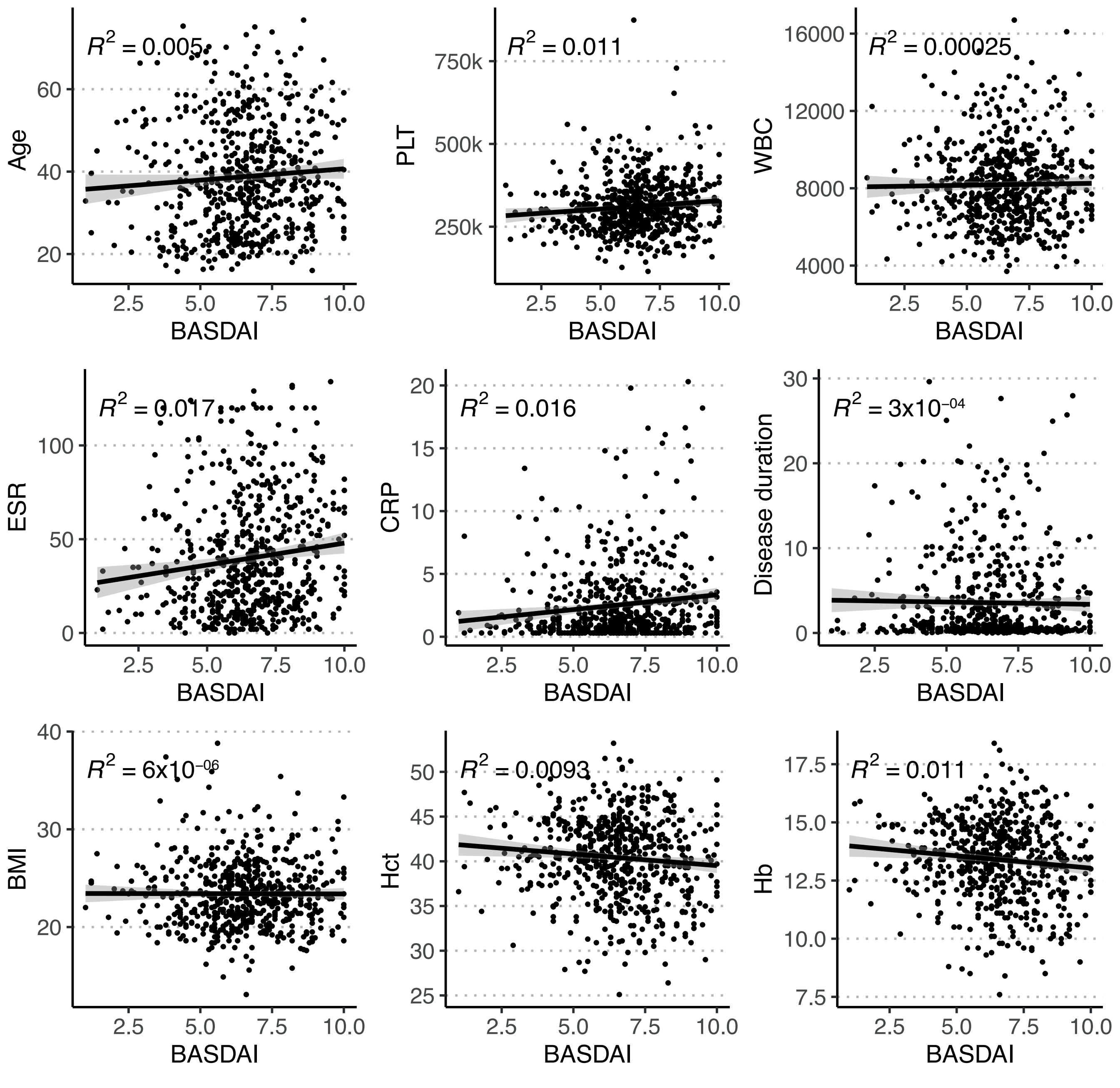

(e) PtGA and other features in patients with AS

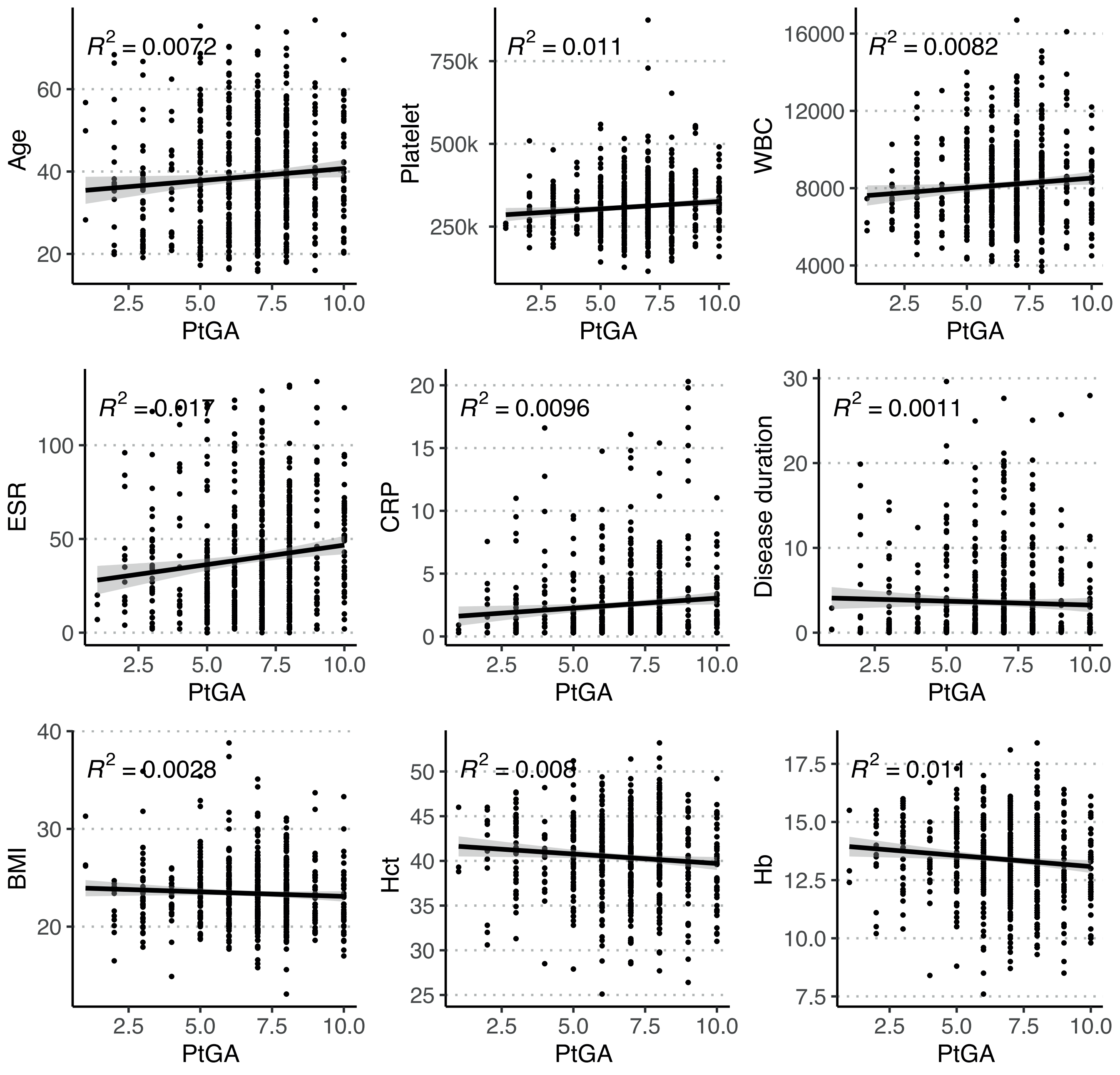

(f) between patient self-reporting scales in both diseases.

RA

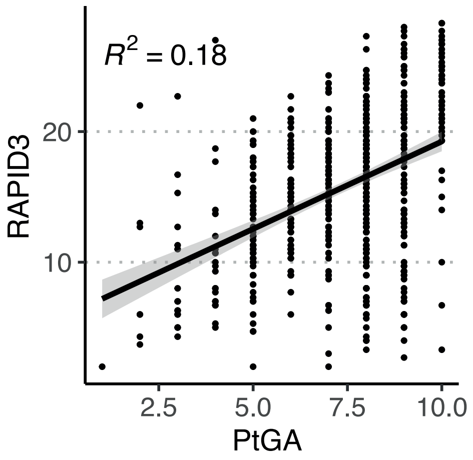

AS

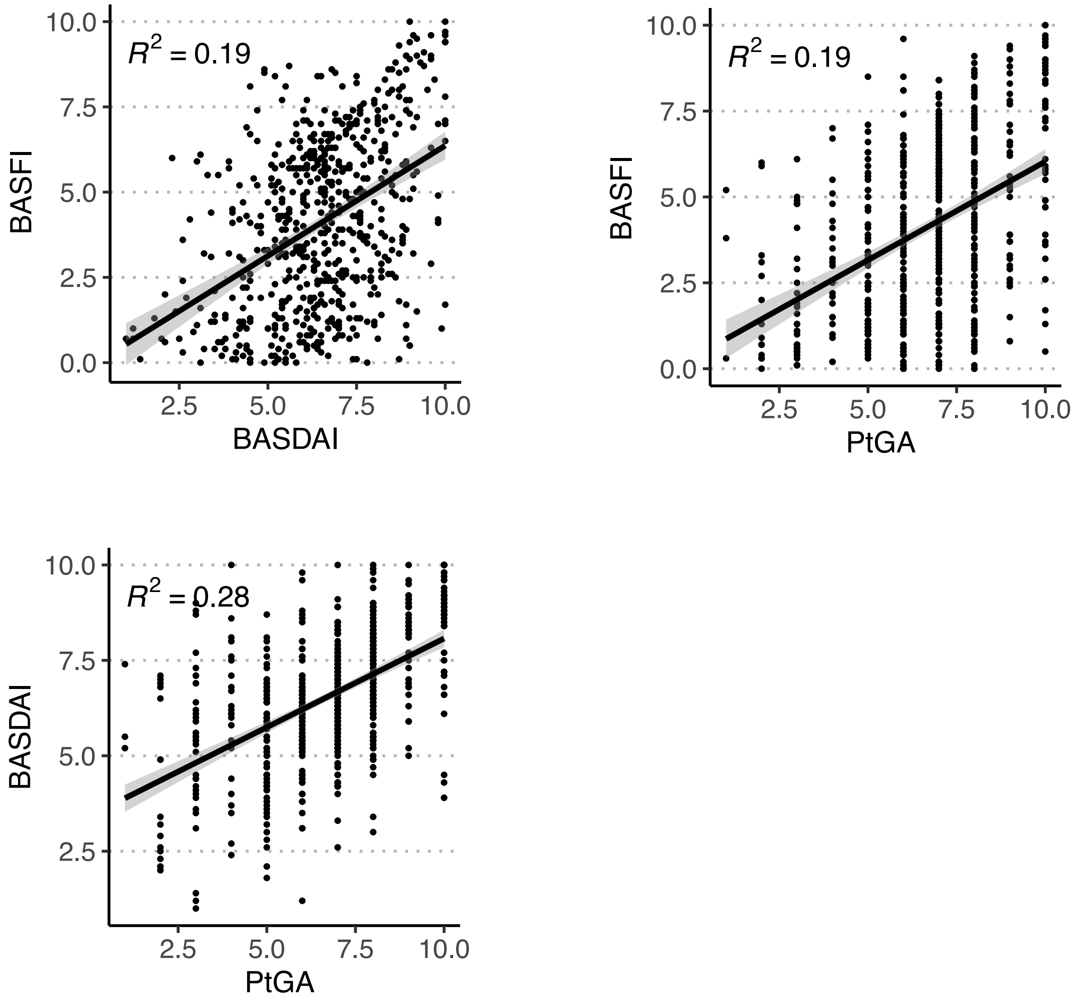

Figure S13. Prediction performances of logistic regression models using patient self-reported scales and their combinations. (a) RA, (b) AS.

(a) RA

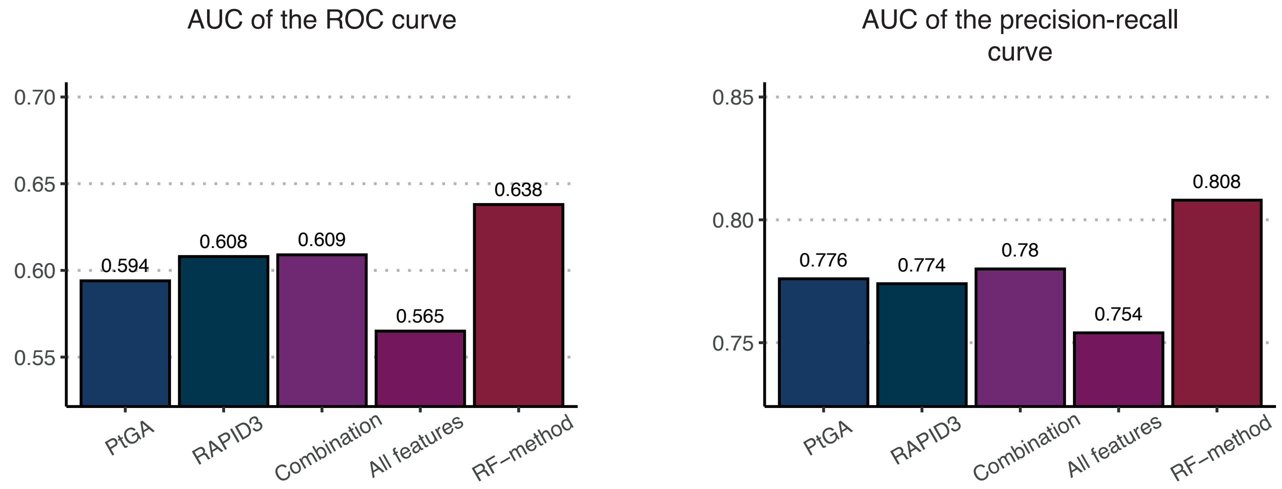

(b) AS

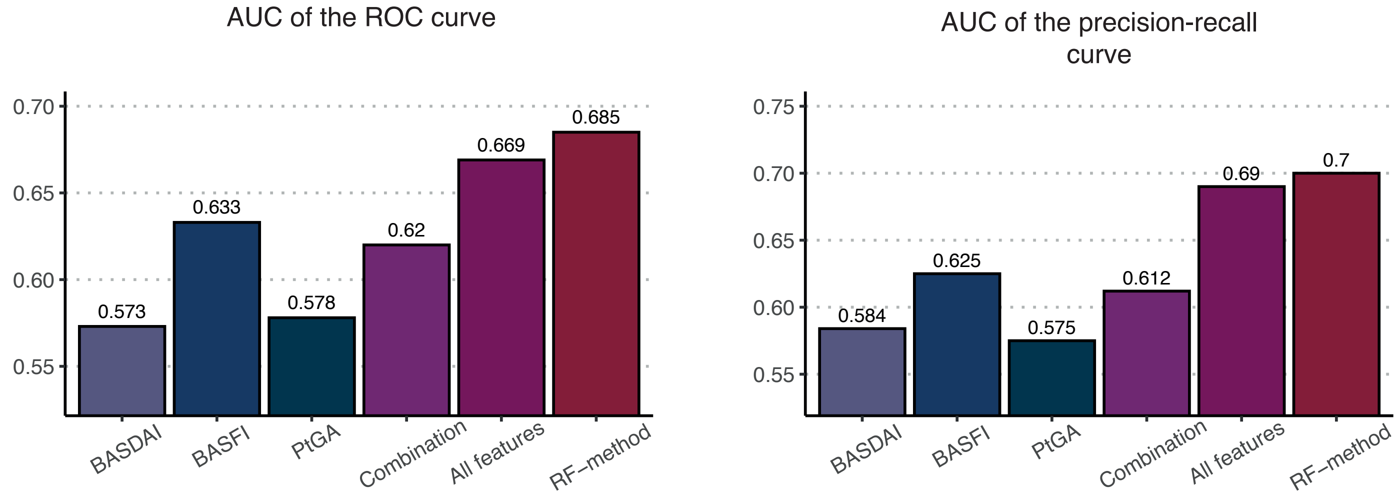

Supplement: Supplementary file 2 — Additional file 2: Figure S1-3. Prediction performance of machine learning methods by varying hyperparameters in RA patients. Figure S4-6. Prediction performance of machine learning methods by varying hyperparameters in AS patients. Figure S7. Performance of RF-method and logistic regression models in RA patients divided by the type of bDMARDs. Figure S8. Result of the feature importance analysis of the best-performing RF-method model in RA patients divided by the type of bDMARDs. Figure S9. Performance of RF-method and logistic regression models in AS patients divided by the type of bDMARDs. Figure S10. Feature importance analysis of the best-performing RF-method model in AS patients divided by the type of bDMARDs. Figure S11. Correlation analysis between input features that reported the feature importance analysis. Figure S12. Linear regression analysis with dot plot results for input features. Figure S13. Prediction performances of logistic regression models using patient self-reported scales and their combinations. [file 13075_2021_2635_MOESM2_ESM.pdf]
