# Supplementary material for: Machine learning-based prediction model for responses of bDMARDs in patients with rheumatoid arthritis and ankylosing spondylitis
Source: Arthritis Res Ther. 2021 Oct 9;23:254. doi: 10.1186/s13075-021-02635-3 (PMC8501710; doi:10.1186/s13075-021-02635-3)
Supplement: Supplementary file 3 — Additional file 3: Table S1. List of input features. Table S2. Chosen hyperparameter sets for each model. [file 13075_2021_2635_MOESM3_ESM.docx]

| **Features** | **Category^a^** | **Features** | **Category** |
| --- | --- | --- | --- |
| anti-CCP antibody | 3 | Glomerulonephritis | 7 |
| Age | 4 | RA related lung disease | 7 |
| BMI | 4 | Pleuritis | 7 |
| Anemia | 6 | ESR | 3 |
| Arrhythmia | 6 | Rheumatoid nodule | 7 |
| Asthma | 6 | Sjogren syndrome | 7 |
| Heart failure | 6 | Sex | 4 |
| COPD | 6 | Hb | 3 |
| Depression | 6 | Hct | 3 |
| Dyslipidemia | 6 | History of joint replacement | 6 |
| HBV | 6 | History of osteoporotic fracture | 6 |
| Hyperthyroidism | 6 | Latent TBc | 8 |
| Hypothyroidism | 6 | Previous use of bucillamine | 5 |
| Hyptertension | 6 | Previous use of hydroxychloroquine | 5 |
| Smoking | 4 | PhGA | 8 |
| Ischemic heart disease | 6 | Previous use of leflunomide | 5 |
| Liver disease | 6 | Platelet | 3 |
| Obesity | 6 | Previous use of methotrexate | 5 |
| Other neurologic disease | 6 | Previous use of sulfasalazine | 5 |
| Steroid use at baseline | 6 | Previous use of tacrolimus | 5 |
| Osteoporosis | 6 | PtGA | 1 |
| Psychosis | 6 | RAPID3 | 1 |
| Peptic ulcer | 6 | Rheumatoid factor | 3 |
| ILD | 6 | Concomitant use of csDMARDs | 5 |
| Renal failure | 6 | SJC | 2 |
| CRP | 3 | TJC | 2 |
| Stroke | 6 | Disease duration | 8 |
| TBc | 6 | WBC | 3 |
| Weight loss | 6 | Abatacept | 5 |
| DM | 6 | Adalimumab | 5 |
| Bucillamine | 5 | Etanercept | 5 |
| Hydroxychloroquine | 5 | Golimumab | 5 |
| Leflunomide | 5 | Infliximab | 5 |
| Methotrexate | 5 | Tocilizumab | 5 |
| csDMARDs use at baseline | 5 | Tofacitinib | 5 |
| Sulfasalazine | 5 | Headache | 6 |
| Tacrolimus | 5 | Malignancy | 6 |
| Category^a^  1: Patient self-reporting scales  2: Physical examination  3: Laboratory results  4: Demographics  5: Medication use  6: Comorbidities  7: Extra-articular features  8: Others  CCP, cyclic citrullinated peptide; BMI, body mass index; COPD, chronic obstructive pulmonary disease; HBV, hepatitis B virus; ILD, interstitial lung disease; CRP, C-reactive protein; TBc, tuberculosis; DM, diabetes mellitus; csDMARDs, conventional synthetic disease-modifying anti-rheumatic drugs; ESR, erythrocyte sediment rate; Hb, hemoglobin; Hct, hematocrit; PtGA, Patient global assessment of disease activity; RAPID3, routine assessment of patient index data 3; SJC, swollen joint count; TJC, tender joint count; WBC, white blood cell. | | | |

**Table S1** List of input variables

**(a) Rheumatoid arthritis**

**(b) Ankylosing spondylitis**

| **Features** | **Category^a^** | **Features** | **Category** |
| --- | --- | --- | --- |
| Age | 4 | Dactylitis | 7 |
| BASDAI | 1 | DM | 6 |
| BASFI | 1 | Sacroiliitis | 7 |
| No NSAIDs use | 5 | Enthesitis | 7 |
| Aceclofenac | 5 | ESR | 3 |
| Celecoixb | 5 | Sex | 4 |
| Etodolac | 5 | Hb | 3 |
| Meloxicam | 5 | Hct | 3 |
| Morniflumate | 5 | History of joint replacement | 6 |
| Nabumetone | 5 | HLA-B27 | 3 |
| Naproxen | 5 | History of osteoporotic fracture | 6 |
| Nimesulide | 5 | IBD | 7 |
| Other NSAIDs | 5 | Latent TBc | 9 |
| Pelubiprofen | 5 | History of peripheral arthritis | 7 |
| Talniflumate | 5 | Peripheral arthritis at baseline | 7 |
| Zaltoprofen | 5 | Plt | 3 |
| BMI | 4 | Psoriasis | 7 |
| Alcohol | 6 | PtGA | 1 |
| Anemia | 6 | No csDMARDs use | 5 |
| Arrhythmia | 6 | Methotrexate | 5 |
| Asthma | 6 | Sulfasalazine | 5 |
| Heart failure | 6 | Family history of AS | 7 |
| COPD | 6 | Response to NSAIDs | 7 |
| Dyslipidemia | 6 | Inflammatory back pain | 7 |
| HBV | 6 | SJC | 2 |
| Hyperthyroidism | 6 | TJC | 2 |
| Hypothyroidism | 6 | Disease duration | 9 |
| Hyptertension | 6 | Uveitis | 7 |
| Smoking | 4 | WBC | 3 |
| Ischemic heart disease | 6 | Sacroliliitis on X-ray, Left | 8 |
| Liver disease | 6 | Sacroliliitis on X-ray, Right | 8 |
| Obesity | 6 | Adalimumab | 51 |
| Other neurologic disease | 6 | Etanercept | 51 |
| Osteoporosis | 6 | Golimumab | 51 |
| Peptic ulcer | 6 | Infliximab | 51 |
| ILD | 6 | Headache | 6 |
| CRP | 3 | Malignancy | 6 |
| Weight loss | 6 |  |  |
| Category^a^  1: Patient self-reporting scales  2: Physical examination  3: Laboratory results  4: Demographics  5: Medication use  6: Comorbidities  7: Extra-articular features  8: Imaging  9: Others  BASDAI, Bath ankylosing spondylitis disease activity index; BASFI, Bath ankylosing spondylitis functional index; NSAIDs, nonsteroidal anti-inflammatory drug; BMI, body mass index; COPD, chronic obstructive pulmonary disease; HBV, hepatitis B virus; ILD, interstitial lung disease; CRP, C-reactive protein; DM, diabetes mellitus; ESR, erythrocyte sediment rate; Hb, hemoglobin; Hct, hematocrit; HLA, human leukocyte antigen; IBD, inflammatory bowel disease; TBc, tuberculosis; Plt, platelet; PtGA, Patient global assessment of disease activity; csDMARDs, conventional synthetic disease-modifying anti-rheumatic drugs; AS, ankylosing spondylitis; SJC, swollen joint count; TJC, tender joint count; WBC, white blood cell. | | | |

| **Methods** | **Hyperparameters** | **RA** | **AS** |
| --- | --- | --- | --- |
| RF | Number of trees | 1000 | 1000 |
|  | Maximum number of tree depth | 4 | No limitation |
|  | Minimum sample split | 3 | 4 |
|  | Minimum leaf samples | 5 | 2 |
| XGBoost | Learning rate | 0.01 | 0.03 |
|  | Maximum number of tree depth | 9 | 2 |
|  | Gamma | 3 | 0.1 |
| ANN | Number of hidden layers | 1 | 2 |
|  | Number of hidden nodes | 200 | 250 |
|  | Learning rate | 3 × 10^-5^ | 1 × 10^-5^ |

**Table S2** Chosen hyperparameter sets for each model

*RA* rheumatoid arthritis, *AS* ankylosing spondylttis, *RF* random forest, *XGBoost* extreme gradient boosting, *ANN* artificial neural network
